# Brain endothelial tricellular junctions as novel sites for T cell diapedesis across the blood–brain barrier

**DOI:** 10.1242/jcs.253880

**Published:** 2021-04-26

**Authors:** Mariana Castro Dias, Adolfo Odriozola Quesada, Sasha Soldati, Fabio Bösch, Isabelle Gruber, Tobias Hildbrand, Derya Sönmez, Tejas Khire, Guillaume Witz, James L. McGrath, Jörg Piontek, Masuo Kondoh, Urban Deutsch, Benoît Zuber, Britta Engelhardt

**Affiliations:** 1 Theodor Kocher Institute, University of Bern, Bern, Switzerland; 2 Institute of Anatomy, University of Bern, Bern CH-3012, Switzerland; 3 Department of Biomedical Engineering, University of Rochester, Rochester, NY 270168, USA; 4 Microscopy Imaging Center (MIC), University of Bern, Bern CH-3012, Switzerland; 5 Science IT Support (ScITS), Mathematical Institute, University of Bern, Bern CH-3012, Switzerland; 6 Institute of Clinical Physiology, Charité – Universitätsmedizin Berlin, Berlin 10117, Germany; 7 Graduate School of Pharmaceutical Sciences, Osaka University, Osaka 565-0871, Japan

**Keywords:** Blood–brain barrier, Tricellular junctions, T cells, Diapedesis, SBF-SEM

## Abstract

The migration of activated T cells across the blood–brain barrier (BBB) is a critical step in central nervous system (CNS) immune surveillance and inflammation. Whereas T cell diapedesis across the intact BBB seems to occur preferentially through the BBB cellular junctions, impaired BBB integrity during neuroinflammation is accompanied by increased transcellular T cell diapedesis. The underlying mechanisms directing T cells to paracellular versus transcellular sites of diapedesis across the BBB remain to be explored. By combining *in vitro* live-cell imaging of T cell migration across primary mouse brain microvascular endothelial cells (pMBMECs) under physiological flow with serial block-face scanning electron microscopy (SBF-SEM), we have identified BBB tricellular junctions as novel sites for T cell diapedesis across the BBB. Downregulated expression of tricellular junctional proteins or protein-based targeting of their interactions in pMBMEC monolayers correlated with enhanced transcellular T cell diapedesis, and abluminal presence of chemokines increased T cell diapedesis through tricellular junctions. Our observations assign an entirely novel role to BBB tricellular junctions in regulating T cell entry into the CNS.

This article has an associated First Person interview with the first author of the paper.

## Abstract (precis)

**Highlighted Article:** Ultrastructural analysis of T cell migration across the blood–brain barrier (BBB) under physiological flow identifies BBB tricellular junctions as sites of T cell diapedesis.

## INTRODUCTION

Maintenance of central nervous system (CNS) homeostasis is a prerequisite for proper neuronal function and is ensured by the endothelial blood–brain barrier (BBB), which allows for a separation between blood and the neural tissue. Continuous and complex tight junctions between adjacent BBB endothelial cells combined with lack of fenestrations and low pinocytotic activity prohibit uncontrolled paracellular and transcellular diffusion of water-soluble molecules across the BBB (Daneman, 2012; Engelhardt and Sorokin, 2009). Claudin-5, occludin and junctional adhesion molecules (JAMs) compose the BBB tight junctions that represent the core structure that actively seals the paravascular space between CNS microvascular endothelial cells in the brain (Castro Dias et al., 2019b). At the same time, transport of necessary nutrients into the CNS and export of potentially toxic metabolites is ensured by a multitude of specific transporters and enzymes expressed by BBB endothelial cells (Sweeney et al., 2019). The BBB also strictly controls immune cell trafficking into the CNS. In homeostatic conditions, passage of immune cells across the BBB into perivascular or subarachnoid spaces is limited to activated CD4^+^ and CD8^+^ T cells, allowing for CNS immune surveillance (Marchetti and Engelhardt, 2020). However, in neuroinflammation such as in multiple sclerosis (MS) or its animal model, experimental autoimmune encephalomyelitis (EAE), increased numbers of immune cells breach the BBB and infiltrate the CNS parenchyma leading to clinical disease (Sallusto et al., 2012; Engelhardt et al., 2017).

Accounting for the unique tightness of the BBB, multi-step T cell migration across the BBB is characterized by unique adaptations (Marchetti and Engelhardt, 2020). Firm arrest of T cells to the BBB endothelium is mediated by the T-cell integrins LFA-1 (α_L_β_2_ integrin) and VLA-4 (α_4_β_1_ integrin) engaging their endothelial ligands, ICAM-1 and VCAM-1, respectively. Following their arrest, T cells polarize and crawl over extended distances on the BBB endothelium against the direction of blood flow in an ICAM-1- and ICAM-2-dependent manner searching for rare sites permissive for diapedesis (Bartholomäus et al., 2009; Steiner et al., 2010). At the ultrastructual level, T cell crawling can be observed to require the continuous extension and retraction of T cell protrusions into the BBB endothelium (Abadier et al., 2015). T cell diapedesis across the BBB finally occurs either paracellularly, through the endothelial cell junctions, or transcellularly, through the endothelial cell body, via a pore-like structure (Engelhardt and Wolburg, 2004). In contrast to peripheral vascular beds, where leukocyte diapedesis occurs mainly through the endothelial cell junctions (Muller, 2015), the inflamed BBB rather favors transcellular T cell diapedesis, possibly by a caveolin-1-dependent mechanism (Wolburg et al., 2005; Lutz et al., 2017). We have previously shown that under low inflammatory conditions, low brain endothelial cell surface expression of ICAM-1 directs T cells to paracellular sites of diapedesis, whereas high expression levels of endothelial ICAM-1 during exacerbated inflammation prohibit T-cell crawling on the BBB and promote a shift towards transcellular diapedesis (Abadier et al., 2015). As neuroinflammation is accompanied by the loss of BBB tight junction integrity, these findings show that the route of T cell diapedesis across the BBB is regulated independently of mechanisms regulating tight junction integrity. Similarly, absence of PECAM-1 from the BBB cell–cell contacts, which leads to impaired junctional integrity, results in increased transcellular T cell diapedesis across the BBB (Wimmer et al., 2019). Taken together, these observations underscore that the BBB endothelium plays an active role in directing T cells to paracellular or transcellular sites of diapedesis; however, the underlying mechanisms remain incompletely understood.

In this study, we aimed to identify subcellular structures accompanying transcellular versus paracellular T cell diapedesis across the BBB under physiological flow at the ultrastructural level. Combining microfluidics using primary mouse brain microvascular endothelial cells (pMBMECs) with live-cell imaging and serial block-face scanning electron microscopy (SBF-SEM) allowed us to conduct a three-dimensional (3D) ultrastructural analysis of T cell diapedesis across the BBB under low and high inflammatory conditions. With this experimental approach, we made the surprising observation that under low inflammatory conditions T cell diapedesis across pMBMECs preferentially occurs through tricellular junctions. High inflammatory conditions with increased transcellular T cell diapedesis were associated with downregulation of tricellulin (also known as MARVELD2) and lipolysis-stimulated lipoprotein receptor (also known as angulin-1, referred to hereafter as LSR/angulin-1), the molecules forming tricellular junctions of epithelial layers and the BBB. Protein-based targeting of LSR/angulin-1 and claudin-5 in pMBMECs under physiological flow *in vitro* led to reduced paracellular and enhanced transcellular T cell diapedesis, whereas abluminal deposition of inflammatory chemokines directed T cell diapedesis to tricellular junctions. Taken together, the combination of *in vitro* live-cell imaging, 3D ultrastructural analysis and functional assays allowed us to identify BBB tricellular junctions as relevant sites for T cell diapedesis in this vascular bed.

## RESULTS

### Phenotype and barrier characteristics of pMBMECs in non-stimulated and inflammatory conditions

**Fig. 1.**
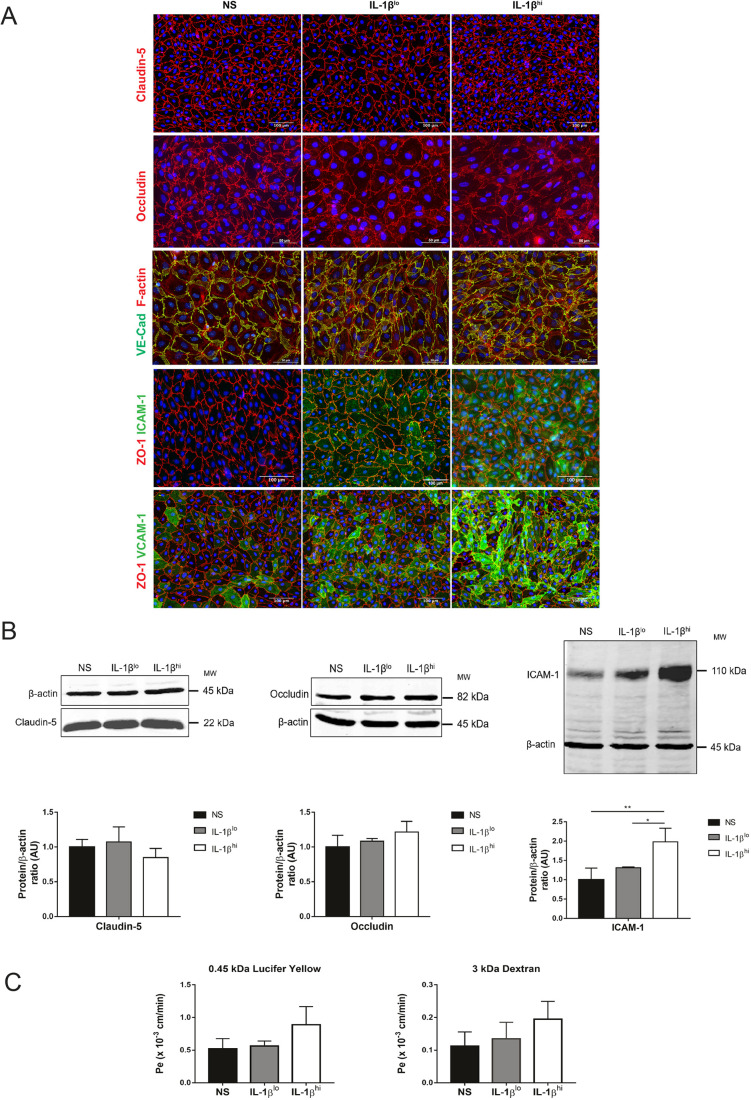
**Phenotype and barrier characteristics of pMBMEC monolayers.** (A) Immunofluorescence staining of non-stimulated (NS), IL-1β^lo^- or IL-1β^hi^-stimulated pMBMEC monolayers. Immunostaining for claudin-5 (red) or occludin (red), F-actin staining (red) in VE-Cadherin–GFP^+^ (green) pMBMECs, and immunostaining for ZO-1 (red) and either ICAM-1 or VCAM-1 (green) are shown. Nuclei are stained with DAPI (blue). Data are representative of three independent experiments. (B) Immunoblot analysis and quantification of claudin-5, occludin and ICAM-1 in non-stimulated, IL-1β^lo^- or IL-1β^hi^-stimulated pMBMECs is shown. β-actin is shown as a loading control used for normalization during quantification. Bar graphs show mean±s.d. of four independent experiments (AU, arbitrary units). *P<0.05; **P<0.01 (one-way ANOVA with a Tukey post hoc test). (C) Permeability of 0.45 kDa Lucifer Yellow and 3 kDa dextran across unstimulated, IL-1β^lo^- or IL-1β^hi^-stimulated pMBMEC monolayers is shown. Endothelial permeability coefficient (Pe) values were calculated as previously described (Steiner et al., 2011). Bar graphs show mean±s.d. of four independent experiments, with triplicates performed per condition.

We made use of our well characterized *in vitro* model of the mouse BBB established from pMBMECs (Coisne et al., 2005). pMBMECs were either non-stimulated or stimulated with 0.05 ng/ml of IL-1β (IL-1β^lo^) or 20 ng/ml of IL-1β (IL-1β^hi^), previously shown to induce low and high cell surface levels of ICAM-1 on the BBB endothelium, favoring paracellular and transcellular diapedesis, respectively (Abadier et al., 2015). First, we confirmed that IL-1β^lo^ and IL-1β^hi^ pMBMECs showed low and high cell surface levels, respectively, of ICAM-1 and VCAM-1, with a characteristic heterogenous expression pattern between the individual brain endothelial cells (Fig. 1A,B), as previously described (Abadier et al., 2015). Although we found a slight increase in non-junctional immunostaining for the tight junction protein occludin and formation of F-actin stress fibers in IL-1β^lo^- and IL-1β^hi^-stimulated pMBMECs versus non-stimulated pMBMECs (Fig. 1A), the junctional localizations of the tight junction protein claudin-5, the adherens junction protein VE-cadherin (also known as CDH5) and the junctional scaffolding protein ZO-1 (also known as TJP1) were found to be unaltered in both non-stimulated and IL-1β-stimulated pMBMECs (Fig. 1A). This suggests that the overall junctional architecture of pMBMECs is still intact under these inflammatory conditions. Quantification of claudin-5 and occludin protein levels confirmed that there were no significant differences between non-stimulated and IL-1β-stimulated pMBMECs (Fig. 1B), although we did notice a trend towards a decrease in claudin-5 protein levels in IL-1β^hi^-stimulated pMBMECs (Fig. 1B). Considering that pro-inflammatory cytokines can induce impaired barrier characteristics of the BBB, we investigated the permeability of pMBMECs to Lucifer Yellow (0.4 kDa) and 3 kDa dextran (Fig. 1C). In accordance with our previous observations (Abadier et al., 2015), we found a visible, although not quite significant, increase in permeability for Lucifer Yellow and for 3 kDa dextran across IL-1β^hi^-stimulated pMBMECs compared to non-stimulated and IL-1β^lo^-stimulated pMBMECs (Fig. 1C). Thus, IL-1β^lo^- and IL-1β^hi^-stimulated pMBMECs showed gradually increased expression of VCAM-1 and ICAM-1 compared to expression in non-stimulated pMBMECs. This was accompanied by a trend towards impaired junctional integrity and higher permeability in IL-1β^hi^ versus IL-1β^lo^ and non-stimulated pMBMEC monolayers.

### The BBB directs T cell diapedesis to tricellular junctions

**Fig. 2.**
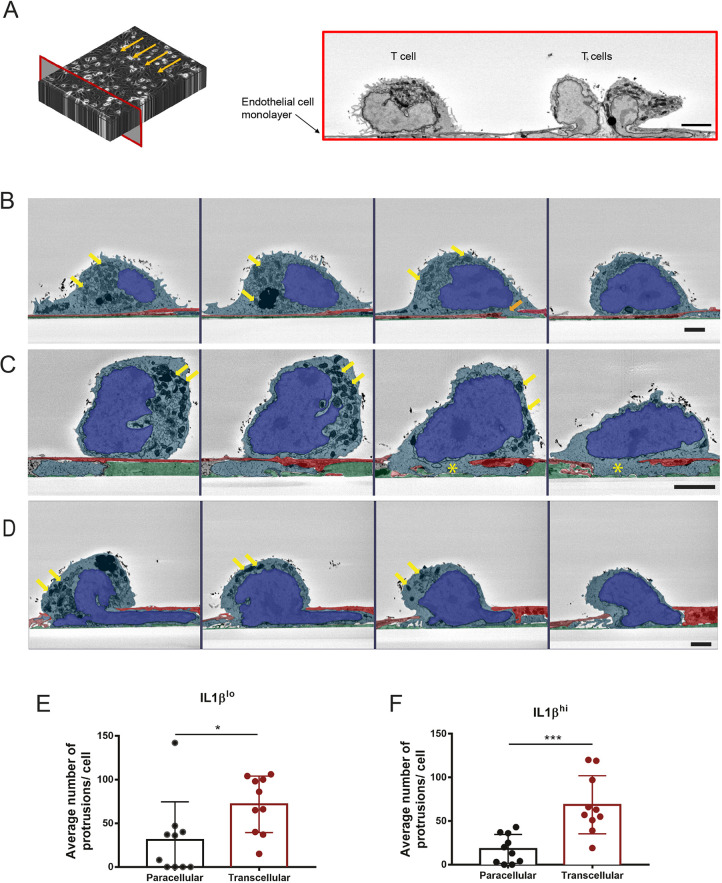
**SBF-SEM of CD4^+^ T_EM_ interacting with pMBMECs under physiological flow.** (A) Scheme of the acquisition made using SBF-SEM (left) and corresponding representative image (right) of this frontal plane (red frame). Image collection was performed precisely against the direction of the flow (arrows). In the example chosen, three CD4^+^ T_EM_ cells interacting with the endothelial monolayer are demonstrated. Scale bar: 1 µm. (B–D) Representative images of three different CD4^+^ T_EM_ cells interacting with the pMBMEC monolayer under flow. For each T cell, four SBF-SEM image sections are shown, depicting the interaction of different frontal section planes of the same cell with the endothelium. Images are false colored to show endothelial cells in red, extracellular matrix in green, T cell cytoplasm in light blue and T cell nuclei in bright blue. In B, four images of the same T cell in close contact with the pMBMEC monolayer are shown. In the first, second and fourth images, no disruption of the pMBMEC monolayer by the T cell is visible. In the third image from the left, the orange arrow marks a T cell protrusion through the pMBMEC monolayer. The organelles of the T cell are visibly concentrated around the nucleus. Yellow arrows point to two exemplary mitochondria. In C, another T cell is sending a subendothelial protrusion across the pMBMEC monolayer, directly visible in sections three and four (highlighted with a yellow asterisk). Yellow arrows point to exemplary mitochondria accumulating at the rear of the T cell. In D, a third T cell is undergoing a diapedesis process, where part of the T cell nucleus is already seen underneath the endothelium. Yellow arrows highlight exemplary mitochondria concentrated at the rear of the T cell. The images were acquired at an angle perpendicular to the physiological flow. Scale bars:1 µm. (E,F) Quantification of the number of protrusions each T cell sent across the junctions or across the endothelial cell body of (E) IL-1β^lo^- or (F) IL-1β^hi^-stimulated pMBMECs. For both datasets, a total of ten T cells were evaluated under IL-1β^lo^ and IL-1β^hi^ stimulation, from four independent samples. Analysis was done by evaluating the SBF-SEM dataset using 3dmod software. Data are presented as mean±s.d. *P<0.05; ***P<0.01 (two-tailed, unpaired Student's *t*-test).

To study subcellular structures involved in transcellular versus paracellular diapedesis of CD4^+^ effector-memory T (T_EM_) cells across the BBB, we combined *in vitro* live-cell imaging with microfluidics and SBF-SEM technology. Activated CD4^+^ T_EM_ cells were perfused over IL-1β^lo^- or IL-1β^hi^-stimulated pMBMECs in a custom-made flow chamber (Coisne et al., 2013) with physiological shear forces, and the interaction was fixed under flow after 13 min, a time point where most of the T cells are in the process of diapedesis across the pMBMECs. Retaining the precise orientation of the direction of flow, the samples were processed for SBF-SEM. Samples were cut and images were collected in 60 nm steps in the perpendicular plane against the direction of flow previously applied in the flow chamber (Fig. 2A). A total of 2000 images of CD4^+^ T_EM_ cells interacting with either IL-1β^lo^- or IL-1β^hi^-stimulated pMBMECs were taken per assay (Fig. 2A). We first observed that organelles in CD4^+^ T_EM_ cells in close contact with the IL-1β^lo^ or IL-1β^hi^ pMBMECs and prior to the initiation of diapedesis were distributed throughout the entire T cell body (Fig. 2B). In contrast, as soon as CD4^+^ T_EM_ cells started diapedesis, as defined by insertion of at least part of the nucleus across the pMBMEC monolayer, most T cell organelles, especially mitochondria, accumulated towards the rear of the T cell and remained in this polarized localization during the diapedesis process (Fig. 2C,D). Furthermore, we observed that the individual CD4^+^ T_EM_ cells send numerous protrusions into and across the pMBMECs prior to insertion of the T cell nucleus across the pMBMEC monolayer, the latter being defined as the definite T cell diapedesis site (Barzilai et al., 2017) (Fig. 2D). In both, IL-1β^lo^- and IL-1β^hi^-stimulated pMBMECs, we observed that CD4^+^ T_EM_ cells inserted a comparable number of protrusions across the pMBMEC monolayers, with a significantly higher number of protrusions found through the endothelial cell body compared to through the endothelial cell junctions (Fig. 2E,F). The formation of T cell protrusions is thus independent of the inflammatory state of the BBB and rather seems to be a T cell-mediated process that allows the T cell to search for sites permissive for diapedesis.

**Fig. 3.**
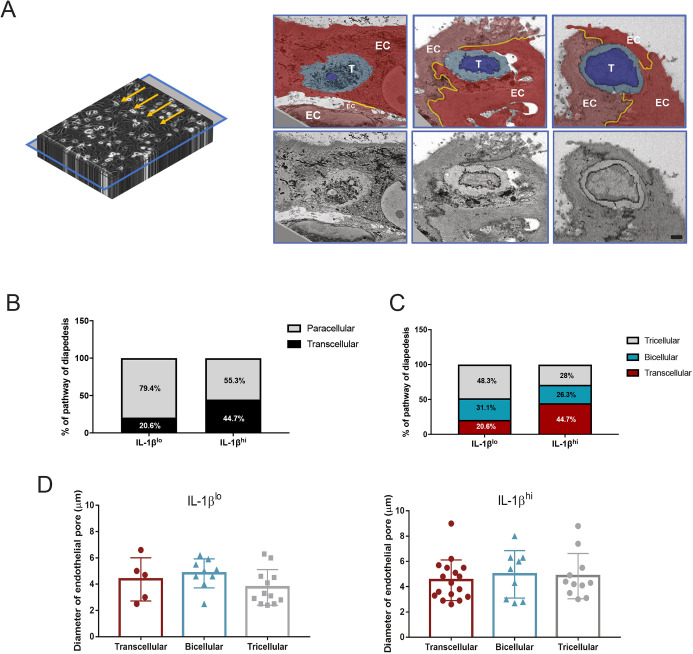
**CD4^+^ T_EM_ cells extravasate across tricellular junctions of the pMBMECs.** (A) Scheme of the acquisition made using SBF-SEM (left) and correspondent representative images (right) of this transversal plane (blue frame). Turning the acquired image stack by 90° allowed us to visualize the precise transversal plane of the interaction between the T cell and the pMBMEC monolayer. The images highlight an example of a transcellular diapedesis (left), an example of a paracellular diapedesis via a bicellular junction (middle) and an example of paracellular diapedesis via a tricellular junction (right). The images in the top row are false colored to show the different endothelial cells in red colors, the T cell cytoplasm in light blue, the T cell nucleus in bright blue and the junctions in yellow. EC, one individual endothelial cell; T, T cell. Original images are shown in the lower row. Scale bar: 1 μm. (B,C) Quantification of the T cell diapedesis pathways observed with SBF-SEM, using 3dmod software. Bar graphs show quantification of either (B) transcellular (black) and paracellular (gray) diapedesis, or (C) transcellular (red), and paracellular diapedesis across bicellular (blue) and tricellular (gray) junctions, across IL-1β^lo^- or IL-1β^hi^-stimulated pMBMECs. For both datasets, we identified a total of 86 CD4^+^ T_EM_ cells interacting with IL-1β^lo^-stimulated pMBMECs and 92 CD4^+^ T_EM_ cells interacting with IL-1β^hi^-stimulated pMBMECs. Of those, a total of 26 and 37 cells were performing diapedesis, respectively, and the pathway of diapedesis was evaluated under IL-1β^lo^ and IL-1β^hi^ conditions from four independent samples. Data were normalized to 100%. (D) Measurement of the endothelial pore diameter generated by CD4^+^ T_EM_ diapedesis through IL-1β^lo^- or IL-1β^hi^-stimulated pMBMECs, during transcellular diapedesis or paracellular diapedesis across bicellular or tricellular junctions. A total of 26 and 37 T cells were evaluated under IL-1β^lo^ and IL-1β^hi^ stimulation, respectively, from four independent samples. Analysis was performed by evaluating the SBF-SEM dataset with 3dmod software. Data are presented as mean±s.d. No statistical differences were found with a one-way ANOVA with a Tukey post hoc test.

To visualize the pathway of diapedesis, we turned our image blocks through 90° in order to look at a transversal plane precisely at the level where CD4^+^ T_EM_ cells were in contact with pMBMECs (Fig. 3A). To identify cells in diapedesis, we analyzed all T cells that had at least part of their nucleus inserted through the pMBMEC monolayer, as detected in the frontal sections. Using the transversal sections, we categorized CD4^+^ T_EM_ cell diapedesis into paracellular or transcellular routes by determining whether or not the T cell disturbed the visible ultrastructure of the junctions. In agreement with our previous work (Abadier et al., 2015), we observed that in the presence of IL-1β^lo^, most of the cells preferentially extravasated through the pMBMEC junctions, whereas in IL-1β^hi^ conditions more T cells were found to undergo transcellular diapedesis (Fig. 3B). In this context, we asked whether there were differences between the morphology of the nuclei of T cells that were undergoing transcellular compared to those of T cells undergoing paracellular diapedesis. 3D segmentation of these nuclei allowed us to observe that irrespective of the cellular diapedesis pathway, the T cell nuclei assumed very dynamic shapes, characterized by the presence of nuclear lobes (Fig. S1).

To our surprise, we noticed that paracellular diapedesis of CD4^+^ T_EM_ cells was not restricted to bicellular endothelial junctions, but rather often occurred at tricellular contacts, a junctional point where three adjacent endothelial cells meet (Fig. 3C). Differentiating paracellular CD4^+^ T_EM_ cell diapedesis into bicellular versus tricellular diapedesis showed that 60% of CD4^+^ T_EM_ cells crossed the pMBMEC monolayer at tricellular contacts rather than at bicellular junctions under low inflammatory conditions (Fig. 3C).

To verify that T cell diapedesis through tricellular junctions is not a random event, we established a null model in which the probability of transcellular versus paracellular diapedesis through bicellular or tricellular junctions was directly proportional to the frequency of their occurrence in a pMBMEC monolayer. By segmenting the cell boundaries of the pMBMEC monolayers and assigning an increasing radius to the cellular junctions, we were able to calculate the frequency of transcellular, bicellular and tricellular junctional diapedesis events if they were to occur in a random fashion in direct correlation to the number of pixels belonging to each category (Fig. S2). Although this model did not include an estimation of the true thickness of pMBMEC junctions, it allowed us to determine that, even under the assumption of a radius of 6 µm for cellular junctions, the frequency of tricellular events would not reach a fraction of 25% (Fig. S2D). This confirmed that BBB tricellular junctions serve as non-random sites for CD4^+^ T_EM_ cell diapedesis across the BBB, especially under low inflammatory conditions.

Finally, we also determined whether transcellular versus paracellular CD4^+^ T_EM_ cell diapedesis required differentially sized pores within one endothelial cell or between two endothelial cells, respectively. To this end, we measured the diameter of the endothelial pores formed during the CD4^+^ T_EM_ cell diapedesis events. Independently of the inflammatory stimulus and the cellular pathway of T diapedesis, the diameter of the pore formed within one pMBMEC or between two or three pMBMECs was close to 5 µm (Fig. 3D), suggesting that this pore size is a minimal requirement for T cell diapedesis across the BBB irrespective of the absence or presence of inflammation.

### Tricellulin and LSR/angulin-1 are expressed in pMBMECs and are downregulated under inflammatory conditions

**Fig. 4.**
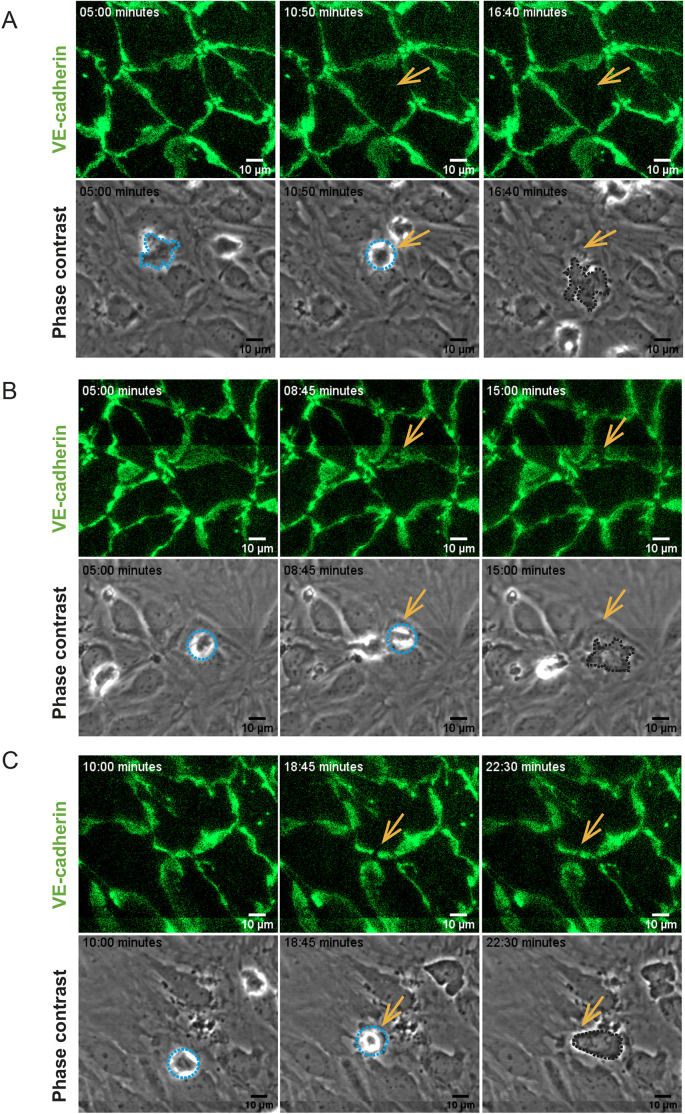
**CD4^+^ T_EM_ cell diapedesis pathways across VE-cadherin–GFP pMBMECs.** Image series from time-lapse videos showing examples of transcellular diapedesis (A) or paracellular diapedesis across bicellular junctions (B) and tricellular junctions (C) over time. The top rows show VE-cadherin–GFP at the endothelial junctions. The bottom rows show the same field of view in phase-contrast images, highlighting CD4^+^ T_EM_ cells interacting with the pMBMECs. T cell diapedesis sites are indicated by arrows, allowing for direct comparison of the absence or presence of a change in the VE-cadherin–GFP pattern with the behavior of the T cells on the pMBMEC monolayer. Junctional migration is visible as a gap in the junctional GFP signal. T cells are highlighted with the dashed lines, where light blue lines show T cells localized on top of the endothelium, whereas dark lines show a T cell below the pMBMEC monolayer. The relative time of image acquisition in minutes is indicated on the top left in each image. Image acquisition over the entire pMBMEC monolayer was divided into eight tiles, which were subsequently stitched together for analysis over the entire field of view. Lines visible in B and C result from the stitching process used to join the individual image tiles.

**Fig. 5.**
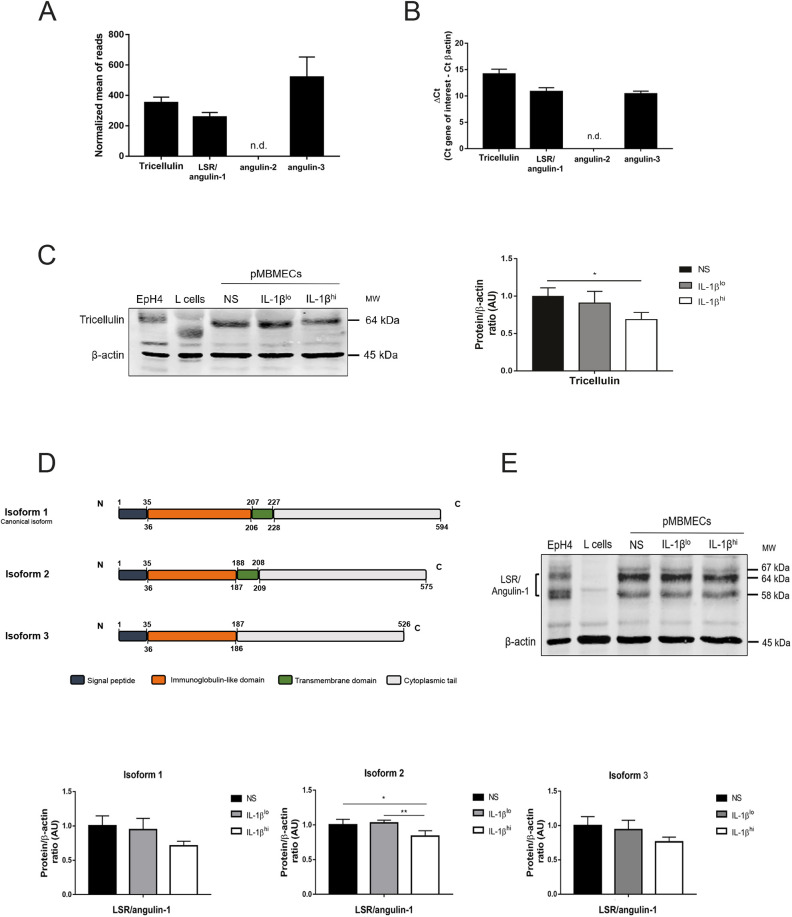
**Components of the tricellular junctions are downregulated upon inflammatory conditions.** (A) Normalized values of read counts for tricellulin and LSR/angulin-1 from an RNA sequencing analysis of non-stimulated pMBMECs, from five independent samples pooled from ten mice each. A threshold of 100 was established for the normalized reads, above which all transcripts were considered as expressed. Data are presented as mean±s.d. (B) Gene expression of tricellulin, LSR/angulin-1, angulin-2 and angulin-3 in non-stimulated pMBMECs was assessed by RT-qPCR. Relative quantification is represented by the ΔCT value (average CT value of target gene−average CT value of β-actin). Data are presented as mean±s.d. of three experiments. (C) Immunoblot analysis and quantification of the expression of tricellulin in non-stimulated (NS), IL-1β^lo^- or IL-1β^hi^-stimulated pMBMECs. EpH4 lysates and L cell lysates were used as positive and negative controls, respectively. β-actin is shown as a loading control used for normalization during quantification. Bar graphs show the mean±s.d. of three independent experiments. *P<0.05 (one-way ANOVA with a Tukey post hoc test). (D) Schematic representation of the isoforms of mouse LSR/angulin-1 and its domains. Numbers indicate the amino acid residues that comprise each domain. (E) Immunoblot analysis (top) and quantification (bottom) of the expression of LSR/angulin-1 isoforms (isoform 1, 67 kDa; isoform 2, 64 kDa; and isoform 3, 58 kDa) in unstimulated, IL-1β^lo^- or IL-1β^hi^-stimulated pMBMECs. EpH4 lysates and L cell lysates were used as positive and negative controls, respectively. β-actin is shown as a loading control used for normalization during quantification. Bar graphs show the mean±s.d. of four independent experiments. *P<0.05; **P<0.01 (one-way ANOVA with a Tukey post hoc test). AU, arbitrary units; n.d., not detected.

Based on our observations using SBF-SEM, it seemed that under low inflammatory conditions CD4^+^ T_EM_ cells preferentially crossed the pMBMEC monolayers via tricellular junctions. We decided to confirm this observation by performing *in vitro* live-cell imaging of activated CD4^+^ T_EM_ cells interacting with IL-1β^lo^- or IL-1β^hi^-stimulated pMBMECs isolated from VE-cadherin–GFP C57BL/6J mice under physiological flow conditions. Identification of pMBMEC junctions based on their GFP signal allowed us to confirm that under low inflammatory conditions CD4^+^ T_EM_ cells cross the pMBMEC monolayer preferentially via tricellular junctions (Fig. 4 and Fig. 8A). In an attempt to understand the signals directing CD4^+^ T_EM_ cells to tricellular contacts, we next wanted to confirm which proteins are specifically localized at tricellular contacts in the pMBMEC monolayers. Tricellulin and LSR/angulin-1 are specifically expressed in brain endothelial cells and localize to tricellular BBB endothelial junctions (Sohet et al., 2015; Iwamoto et al., 2014). Thus, we first asked whether tricellulin and the angulin proteins are expressed in pMBMECs, whether they are localized to tricellular contacts, and whether their expression is affected by inflammation. RNAseq transcriptome analysis (Fig. 5A; Castro Dias et al., 2019a) and RT-qPCR (Fig. 5B) confirmed mRNA expression of tricellulin, LSR/angulin-1 and angulin-3 (also known as ILDR2), but not angulin-2 (also known as ILDR1), in pMBMECs. To confirm tricellular localization of tricellulin and LSR/angulin-1, we performed immunostaining of tricellulin and LSR/angulin-1 on pMBMEC monolayers and on monolayers of the epithelial EpH4 cell line, as a positive control (Masuda et al., 2011; Ikenouchi et al., 2005). Although we readily detected tricellular localization of both proteins in epithelial monolayers, we could not observe any positive immunostaining of pMBMECs using either polyclonal or monoclonal mouse anti-tricellulin and anti-LSR/angulin-1 antibodies (Fig. S3 and data not shown). Protein expression of tricellulin and LSR/angulin-1 in pMBMECs could, however, be confirmed by western blotting (Fig. 5C,E).

Next, we quantified the expression levels of tricellulin and LSR/angulin-1 proteins in pMBMECs in the context of inflammation. As a positive control we used epithelial cell lysates from EpH4 cells, and as a negative control we used lysates from L cells, a fibroblast cell line (Furuse et al., 1999). We found that IL-1β^hi^- but not IL-1β^lo^-stimulated pMBMECs showed downregulated expression of tricellulin (Fig. 5C). With respect to LSR/angulin-1, three different splice variants are described in the mouse genome: isoform 1, isoform 2 and isoform 3 (Higashi et al., 2013; Fig. 5D). Isoform 1 is considered the canonical form and is translated to a full-length protein (67 kDa band). Isoform 2 lacks part of the immunoglobulin-like domain (64 kDa band), and isoform 3 does not possess the transmembrane domain (58 kDa band) (Fig. 5D). Their respective roles in forming or maintaining tricellular junctions is still unknown. Using an antibody that detects the N-terminal part shared by the three LSR/angulin-1 isoforms we could detect expression of isoform 1 and 2, with band sizes between 64 and 67 kDa, as well as isoform 3 as a 58 kDa band in lysates from pMBMECs (Fig. 5E), in accordance with previous observations by others (Sohet et al., 2015). Under IL-1β^hi^ but not under IL-1β^lo^ stimulation of pMBMECs, all LSR/angulin-1 isoforms showed a trend towards a downregulation, with isoform 2 being significantly downregulated (Fig. 5E). Downregulated protein expression of tricellulin and LSR/angulin-1 in IL-1β^hi^-stimulated pMBMECs may thus lead to impaired stabilization of BBB tricellular and bicellular junctions, as previously observed for epithelial cells (Ikenouchi et al., 2005; Krug et al., 2009; Masuda et al., 2011; Sohet et al., 2015).

### Targeting LSR/angulin-1 reduces paracellular CD4^+^ T_EM_ cell diapedesis via bicellular rather than tricellular junctions

**Fig. 6.**
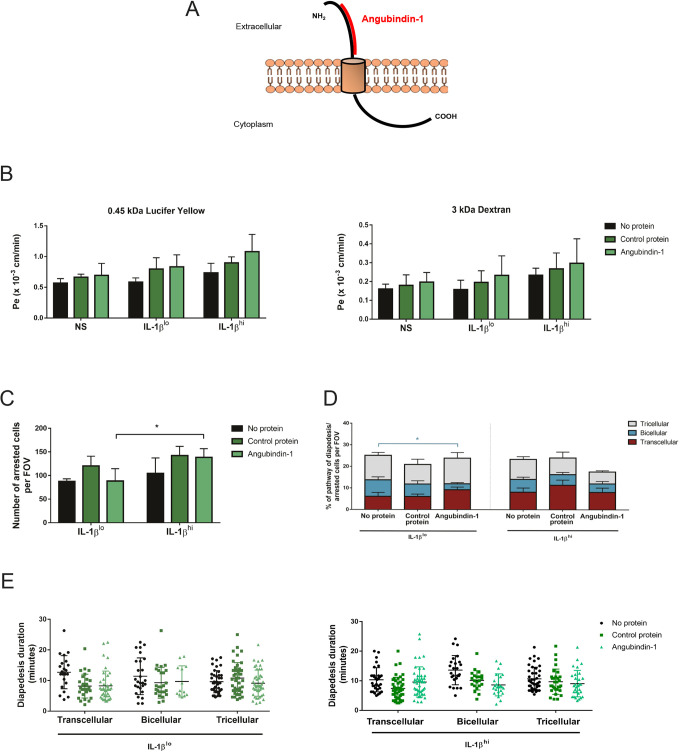
**Targeting of LSR/angulin-1 by angubindin-1 in pMBMECs decreases paracellular CD4^+^ T_EM_ cell diapedesis at bicellular junctions.** (A) Schematic representation of the structure of LSR/angulin-1 and the binding site of angubindin-1 in its N-terminal region. The image was adapted from Servier Medical Art (http://smart.servier.com/), under the terms of a CC-BY 3.0 license. (B) Permeability of differently IL-1β-stimulated pMBMEC monolayers (NS, non-stimulated) to 0.45 kDa Lucifer Yellow (left) and 3 kDa dextran (right), in the presence or absence of the control protein and angubindin-1, as indicated. Endothelial permeability coefficient (Pe) values were calculated as previously described (Steiner et al., 2011). Bar graphs show mean±s.d. of three independent experiments, with three replicates per condition. (C) Mean number of arrested CD4^+^ T_EM_ cells in IL-1β^lo^- or IL-1β^hi^-stimulated endothelium, per field of view (FOV), while targeting LSR/angulin-1 with angubindin-1 as indicated. Bar graphs show mean±s.d. of three experiments. *P<0.05 (one-way ANOVA with a Tukey post hoc test). (D) Quantification of transcellular (red), bicellular junctional (blue) and tricellular junctional (gray) diapedesis events of CD4^+^ T_EM_ cells across IL-1β^lo^- or IL-1β^hi^-stimulated pMBMECs in the presence or absence of the control protein and angubindin-1, as indicated. Both IL-1β and protein stimulations were performed simultaneously for 16 h. In each condition, 100 diapedesis events were evaluated and normalized to the respective number of arrested CD4^+^ T_EM_ cells per FOV, from at least four videos from three independent experiments. Stacked bar graphs show mean±s.d. *P<0.05 (one-way ANOVA with a Tukey post hoc test). (E) Duration of CD4^+^ T_EM_ cell diapedesis through transcellular, bicellular junctional or tricellular junctional pathways, across IL-1β^lo^- or IL-1β^hi^-stimulated pMBMECs, in the presence or absence of the control protein and angubindin-1, as indicated. Each data point represents an individual CD4^+^ T_EM_ cell. The duration of the diapedesis of 100 cells was evaluated per condition, from a total of three independent experiments. Individual data points are plotted, with mean and s.d. indicated.

Downregulated expression of tricellulin and LSR/angulin-1 in highly inflamed pMBMECs correlated with increased transcellular T cell diapedesis. Because we have previously observed that altered molecular composition of endothelial bicellular junctions shifts CD4^+^ T_EM_ cell diapedesis across the BBB to transcellular sites (Wimmer et al., 2019), we next asked whether targeting tricellular junctional proteins would favor transcellular T cell diapedesis across pMBMECs. To this end we used angubindin-1, which consists of amino acids 421–664 of iota-toxin Ib (also known as Ibp) from *Clostridium perfringens*. Angubindin-1 binds to the N-terminal part of LSR/angulin-1 as a function blocking probe (Fig. 6A) and has previously been shown to remove LSR/angulin-1 from the tricellular tight junctions (Krug et al., 2017). As a negative control, we used recombinant C2 protein, corresponding to amino acids 592–721 of C2 toxin of *Clostridium botulinum* (Krug et al., 2017).

We first investigated whether angubindin-1 would impact on the barrier characteristics of pMBMECs. IL-1β^lo^- or IL-1β^hi^-stimulated pMBMECs were incubated during stimulation with either no protein, a control protein or with angubindin-1. Although angubindin-1 has previously been found to increase the permeability of epithelial layers (Krug et al., 2017), we did not observe an angubindin-1-induced change in the endothelial permeability to 0.45 kDa Lucifer Yellow or 3 kDa dextran when compared that of the control cells (Fig. 6B). Moreover, we did not see any angubindin-1-induced effects on expression and location of claudin-5, ZO-1, ICAM-1, VCAM-1 and VE-cadherin (Figs S4, S5 and data not shown). As a next step, we investigated the effect of angubindin-1 on the cellular pathway of T cell diapedesis across pMBMECs. Following incubation with control peptide or angubinding-1, we imaged the interaction of CD4^+^ T_EM_ cells with IL-1β^lo^- and IL-1β^hi^-stimulated VE-cadherin-GFP^+^ pMBMECs under physiological flow (Fig. 4). The number of arrested CD4^+^ T_EM_ cells on the pMBMEC monolayers was comparable between the control protein- and angubindin-1-treated monolayers (Fig. 6C,D). Surprisingly, when the pMBMECs were incubated with angubindin-1, the number of arrested T cells on IL-1β^hi^-stimulated pMBMECs was increased compared to the number of arrested T cells on IL-1β^lo^-stimulated monolayers (Fig. 6C). Analysing the cellular pathway of T cell diapedesis, we confirmed our earlier observations that under IL-1β^lo^ stimulation the majority of T cells crossed the pMBMEC monolayer via the paracellular pathway, whereas under IL-1β^hi^ stimulation transcellular T cell diapedesis was significantly increased (Fig. 6D and Fig. 8A). Whereas incubation with the control protein did not influence the cellular pathway of T cell diapedesis across pMBMEC monolayers (Fig. 6D), preincubation of IL-1β^lo^-stimulated pMBMECs with angubindin-1 surprisingly reduced the fraction of paracellular CD4^+^ T_EM_ cell diapedesis via bicellular rather than tricellular junctions, when compared to the controls (Fig. 6D). At the same time, treatment with angubindin-1 did not further increase transcellular CD4^+^ T_EM_ cell diapedesis across IL-1β^hi^-stimulated pMBMECs (Fig. 6D). Furthermore, angubidin-1 did not affect the diapedesis duration of T cells across the different diapedesis routes under both IL-1β stimulation conditions (Fig. 6E). Analysis of the T cell crawling distance that preceded diapedesis also demonstrated no differences in the presence of angubindin-1 (data not shown). Taken together, these observations show that targeting LSR/angulin-1 in brain endothelium favors transcellular T cell diapedesis across the endothelium without affecting the endothelial barrier integrity.

### Targeting endothelial claudin-5 decreases paracellular CD4^+^T_EM_ cell diapedesis across bicellular junctions

**Fig. 7.**
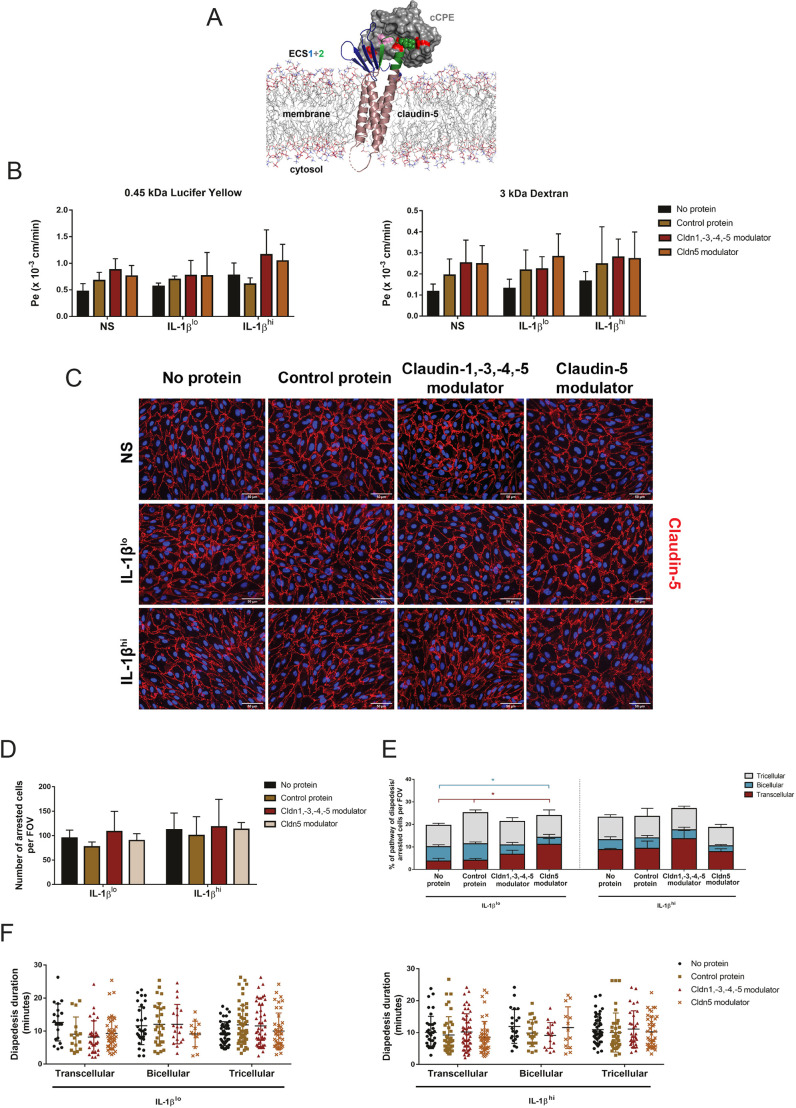
**Protein targeting of claudin-5 in pMBMECs decreases paracellular CD4^+^ T_EM_ cell diapedesis at bicellular junctions.** (A) Schematic model of claudin-5 modulator (cCPE variant) binding to claudin-5 that sterically blocks claudin polymerization. cCPE (gray surface) was mutated at three positions (N218Q, Y306W and S313H; red) to enable binding to claudin-5 [backbone shown as ribbons; transmembrane helices, violet; extracellular segment (ECS) 1, blue; ECS2, green]. N218Q (red, left) facilitates interaction with ECS1, Y306W (red, right) and S313H (red, middle) facilitate interaction with ECS2. Mutation L315A (pink) together with Y306A blocks cCPE interaction with claudins. ECS2 positions critical for interaction are shown as green spheres. Phospholipids of the membrane are shown as lines (head groups in color). Image created using cCPE–claudin-4 crystal structure (PBD ID: 5b2g) and data from Neuhaus et al. (2018), generated with Maestro and Pymol (Schrödinger). (B) Permeability of differently stimulated pMBMEC monolayers (NS, non-stimulated) to 0.45 kDa Lucifer Yellow (left) and 3 kDa dextran (right), in the presence or absence of claudin (Cldn) modulator or control cCPE proteins, as indicated. Endothelial permeability coefficient (Pe) values were calculated as previously described (Steiner et al., 2011). Bar graphs show mean±s.d. of four independent experiments, with three replicates per condition. (C) Immunofluorescence staining of claudin-5 (red) in pMBMEC monolayers. Comparable staining was obtained for claudin-5 in either non-stimulated (NS), IL-1β^lo^- or IL-1β^hi^-stimulated pMBMECs, in the presence or absence of the cCPE proteins. Nuclei were stained with DAPI (blue). Three independent experiments were performed. Scale bars: 50 µm. (D) Mean number of arrested CD4^+^ T_EM_ cells in IL-1β^lo^- or IL-1β^hi^-stimulated endothelium, per field of view (FOV), while targeting claudin-5, as indicated. Data are presented as mean±s.d. of three experiments. (E) Quantification of transcellular (red), bicellular (blue) and tricellular (gray) diapedesis events of CD4^+^ T_EM_ cells across IL-1β^lo^- or IL-1β^hi^-stimulated pMBMECs, in the presence or absence of the cCPE peptides. Both IL-1β and claudin modulator stimulations were performed simultaneously for 16 h. In each condition, 100 diapedesis events were evaluated and normalized to the respective number of arrested CD4^+^ T_EM_ cells per FOV, from at least four videos from four independent experiments. Stacked bar graphs show mean±s.d. *P<0.05 (one-way ANOVA with a Tukey post hoc test). (F) Duration of CD4^+^ T_EM_ cell diapedesis through transcellular, bicellular junctional or tricellular junctional pathways, across IL-1β^lo^- or IL-1β^hi^-stimulated pMBMECs, in the presence or absence of the cCPE proteins. Each data point represents an individual CD4^+^ T_EM_ cell. The duration of the diapedesis of 100 cells was evaluated per condition, from a total of four independent experiments. Individual data points are plotted, with mean and s.d. indicated.

To test whether direct modulation of a component of the bicellular junctions would also enhance transcellular T cell diapedesis events, we targeted claudin-5, a bicellular junctional protein that is highly expressed in BBB tight junctions, again using specific recombinant protein inhibitors. The non-toxic C-terminal domain of *Clostridium perfringens* enterotoxin (cCPE) binds to the extracellular segments 1 and 2 (ECS1 and ECS2) of a subset of claudins (Fujita et al., 2000; Veshnyakova et al., 2012; Saitoh et al., 2015) (Fig. 7A). Structure-based mutagenesis has previously been used to generate cCPE variants with altered claudin subtype specificity. These have been used as tight junction modulators that can reversibly open the BBB allowing drug delivery into the CNS (Veshnyakova et al., 2012; Protze et al., 2015; Neuhaus et al., 2018). In the present study, we used two cCPE variants that bind to claudin-5: cCPE Y306W/S313H (referred to hereafter as Cldn1,-3,-4,-5 modulator), which binds to claudin-5 but also to claudin-1, claudin-3 and claudin-4; and cCPE N218Q/Y306W/S313H (referred to hereafter as Cldn5 modulator), which preferentially binds to claudin-5 (Neuhaus et al., 2018). Although claudin-1, -3 and -4 are not expressed in pMBMECs (Castro Dias et al., 2019a), we still opted to use both claudin-5-binding cCPE variants for comparison. As a negative control, we used cCPE Y306A/L315A (referred to hereafter as control protein), which does not recognize any claudin.

We first investigated the permeability of Lucifer Yellow and 3 kDa dextran across IL-1β^lo^- and IL-1β^hi^_-_stimulated pMBMECs, in the presence and absence of control protein, Cldn1, -3, -4, -5 modulator or Cldn5 modulator. We did not observe any effect of the recombinant proteins on permeability of Lucifer Yellow or 3 kDa dextran across the pMBMECs (Fig. 7B). In accordance with previous observations (Neuhaus et al., 2018), the cCPE variants did not affect the bicellular localization of claudin-5 (Fig. 7C), nor did they affect the expression of other junctional and adhesion molecules, such as ZO-1, ICAM-1, VCAM-1 and VE-cadherin (Fig. S6 and data not shown). To investigate whether the recombinant proteins would affect the cellular pathway of CD4^+^ T_EM_ cell diapedesis across pMBMEC monolayers, we isolated pMBMECs from VE-cadherin–GFP mice and investigated CD4^+^ T_EM_ cell interaction with IL-1β^lo^-or IL-1β^hi^-stimulated pMBMEC monolayers under physiological flow by live-cell imaging (Fig. 4). The number of arrested CD4^+^ T_EM_ cells and percentage of diapedesis events on pMBMECs was not affected by recombinant protein pulsing (Fig. 7D,E). We observed the expected ratios of T cell diapedesis pathways with no protein pulsing and in the presence of the control protein, with the majority of CD4^+^ T_EM_ cells preferentially migrating paracellularly under IL-1β^lo^ stimulation, whereas with IL-1β^hi^ stimulation more CD4^+^ T_EM_ cells migrated transcellularly (Fig. 7E). Incubation of IL-1β^lo^-stimulated pMBMECs with the Cldn5 modulator led to a significant decrease of paracellular CD4^+^ T_EM_ cell diapedesis across bicellular junctions, while diapedesis across tricellular junctions was not affected (Fig. 7E). Rather, we observed an increase in transcellular CD4^+^ T_EM_ cell diapedesis events (Fig. 7E). Incubation of IL-1β^lo^-stimulated pMBMECs with the Cldn1, -3, -4, -5 modulator showed similar, however, not quite significant effects compared to those of the Cldn5 modulator (Fig. 7E). Transcellular T cell diapedesis across IL-1β^hi^-stimulated pMBMEC monolayers was not further increased by treatment with the Cldn5 modulator (Fig. 7E).

Finally, we measured the duration of the diapedesis process while targeting claudin-5, to assess whether this allows for a faster paracellular diapedesis. As before, the crawling distance that preceded the diapedesis event and the diapedesis duration across the different diapedesis routes did not differ between the control groups and the conditions targeting claudin-5, under both IL-1β-stimulation conditions (Fig. 7F and data not shown). Taken together, targeting claudin-5 – the main constituent of pMBMEC bicellular tight junctions – under low inflammatory conditions reduced paracellular CD4^+^ T_EM_ cell diapedesis across pMBMECs and increased transcellular diapedesis, with no visible impact on barrier integrity or diapedesis duration.

### Abluminal chemokines increase CD4^+^ T helper 1 cell diapedesis across tricellular junctions

**Fig. 8.**
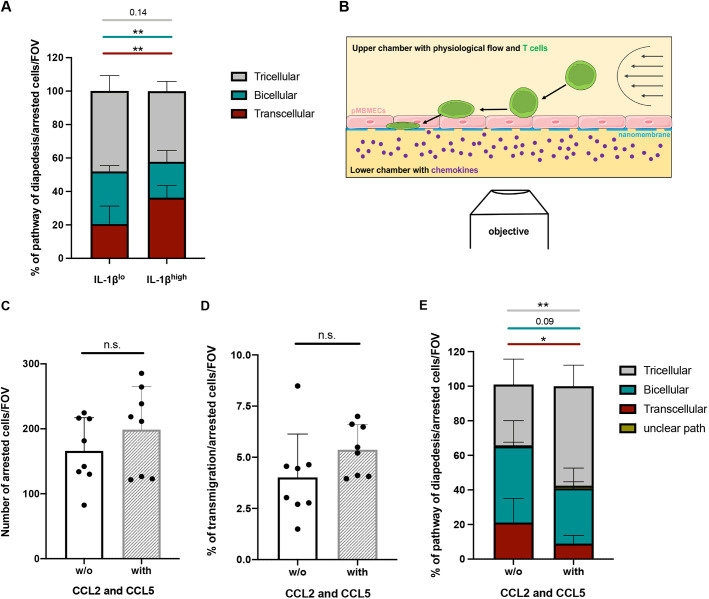
**Abluminal chemokines increase CD4^+^ Th1 cell diapedesis via tricellular junctions of pMBMECs.** (A) Cumulative analysis of transcellular (red), bicellular (teal) and tricellular (gray) diapedesis events of CD4^+^ Th1 cells across IL-1β^lo^- or IL-1β^hi^-stimulated pMBMECs, as shown in Figs 6D and 7E (conditions no protein). In each condition, 100 diapedesis events were evaluated and normalized to the respective number of arrested CD4^+^ Th1 cells per field of view (FOV), from at least four videos from four independent experiments. Stacked bar graphs show mean±s.d. **P<0.01 (one-way ANOVA with a Tukey post hoc test). (B) Schematic representation of *in vitro* live imaging of T cell extravasation across primary mouse brain microvascular endothelial cells (pMBMECs) cultured on nanoporous silicon nitride (NPN) membranes (µSiM-CVB) under physiological flow conditions (from right to left; arrows) with recombinant mouse CCL2 and CCL5 (both 100 ng/ml) in the bottom compartment. (C) Mean number of arrested Th1 cells on IL-1β^hi^-stimulated pMBMECs under physiological flow conditions in the µSiM-CVB assay, in the presence (with) or absence (w/o) of CCL2 and CCL5 in the bottom channel. (D) Mean percentage of transmigrated Th1 cells across IL-1β^hi^-stimulated pMBMECs in the µSiM-CVB assay, in the presence or absence of CCL2 and CCL5. Each data point shown in C,D represents the mean of the two FOVs per movie. (E) Quantification of transcellular (red), bicellular (teal) and tricellular (gray) diapedesis events of Th1 cells across IL-1β^hi^-stimulated pMBMECs in the µSiM-CVB assay. Events with an unclear transmigration path are shown in yellow. Data in C–E are mean±s.d. of three experiments, with at least duplicates for each condition. *P<0.05; **P<0.01; n.s., not significant (two-tailed, unpaired *t*-test).

Our analysis of the limited number of T cell diapedesis events across pMBMECs at the ultrastructural level suggested that T cell diapedesis via tricellular junctions preferentially occurs under low inflammatory conditions (Fig. 3C). However, when comparing the cellular pathway of the several hundred T cell diapedesis events across IL-1β^lo^- and IL-1β^hi^-stimulated VE-cadherin–GFP^+^ pMBMECs in the absence of peptide treatments (Figs 6, 7 and 8A), we found that ∼40% of T cells crossed the pMBMEC monolayers via tricellular junctions irrespective of the inflammatory conditions. In accordance with our previous observations (Abadier et al., 2015), transcellular T cell diapedesis across IL-1β^hi^-stimulated pMBMECs was significantly increased, while paracellular T cell diapedesis across bicellular junctions was significantly reduced, when compared to that across IL-1β^lo^-stimulated pMBMECs (Fig. 8A).

To further explore the role of tricellular junctions in mediating T cell diapedesis across the BBB, we asked whether chemotactic signals delivered from the abluminal to the luminal side of pMBMECs via tricellular junctions would direct T cell diapedesis across tricellular junctions. The inflammatory chemokines CCL2 and CCL5 mediate T cell migration into the CNS during neuroinflammation (Mahad and Ransohoff, 2003; Zang et al., 2000). We therefore *in vitro* polarized CD4^+^ T helper 1 (Th1) cells and tested their chemotactic behavior towards increasing concentrations of CCL2 and CCL5 across a laminin-coated porous filter in a two-chamber assay over 2 h (Fig. S7). Both CCL2 and CCL5 significantly enhanced T cell migration into the lower chamber, reaching a peak at 100 ng/ml for both chemokines (Fig. S7).

To test whether CCL2 and CCL5 could affect the cellular pathway of T cell diapedesis across pMBMECs under physiological flow, we cultured pMBMECs on nanoporous silicon nitride (NPN) membranes in a two-chambered µSiM-CVB microfluidic device (Fig. 8B; Mossu et al., 2019). CCL2 and CCL5 were added at a concentration of 100 ng/ml to the bottom channel of the µSiM device (i.e. at the basolateral side of IL-1β^hi^-stimulated pMBMECs) 2 h prior to *in vitro* live-cell imaging of T cell interaction with the pMBMEC monolayer under physiological flow (Fig. 8B). The presence of CCL2 and CCL5 neither affected the numbers of Th1 cells arresting on, nor the number of Th1 cells crossing, the pMBMEC monolayers under physiological flow (Fig. 8C,D). At the same time, addition of CCL2 and CCL5 to the bottom channel of the µSiM microfluidic device significantly increased T cell diapedesis via tricellular junctions and reduced transcellular T cell diapedesis across IL-1β^hi^-stimulated pMBMECs (Fig. 8D). Taken together, these observations suggest that BBB tricellular junctions may provide a scaffold for chemokine gradients allowing the guidance of T cell diapedesis to these unique junctional sites of the BBB.

## DISCUSSION

In this study, we identify brain microvascular tricellular junctions as important gateways for controlling T cell diapedesis across the BBB under low and high inflammatory conditions. The molecular composition and potential structure of tricellular junctions has mainly been investigated in epithelial cells. There, the most apical tight junction strands of the bicellular junctions meet at tricellular junctions and continue basally, forming a vertical tube-like structure referred to as the central sealing element (Staehelin et al., 1969). While bicellular tight junctions are mainly composed of claudins, occludin and JAMs (Tietz and Engelhardt, 2015), tricellular junctions harbor tricellulin and members of the angulin family of proteins (Furuse et al., 2014). Tricellulin is a member of the tight junction-associated MARVEL protein family and is structurally similar to occludin, with four transmembrane segments (Ikenouchi et al., 2005). LSR/angulin-1 is the best characterized protein of the angulin family and has an extracellular immunoglobulin-like domain and one single-pass transmembrane domain (Masuda et al., 2011; Higashi et al., 2013). Tricellulin is necessary for the correct morphology of tricellular junctions and maintenance of epithelial barrier properties (Ikenouchi et al., 2005; Nayak et al., 2013). Lack of tricellulin in epithelial cells promotes abnormal subcellular distribution of occludin and increased paracellular permeability, suggesting that tricellulin contributes to maintaining the integrity of epithelial bicellular junctions (Ikenouchi et al., 2005; Krug et al., 2009). LSR/angulin-1 also regulates epithelial barrier integrity and recruits tricellulin to tricellular junctions (Masuda et al., 2011). The precise molecular architecture of tricellular junctions is unknown, but epithelial tricellular junctions are modeled with angulins at the core of the central sealing element, associated with tricellulin, which engages claudins and thus connects to bicellular junctions (Ikenouchi et al., 2008; Cording et al., 2013; Masuda et al., 2011).

Recent studies have found tricellulin and LSR/angulin-1 transcripts specifically enriched in brain endothelial cells (Daneman et al., 2010; Vanlandewijck et al., 2018). Localization of tricellulin and LSR/angulin-1 protein in tricellular contacts of BBB and blood–retinal barrier endothelium (Mariano et al., 2013; Iwamoto et al., 2014; Sohet et al., 2015), but not in vascular beds lacking barrier formation, such as the fenestrated vessels of the choroid plexus and the circumventricular organs, or in the peripheral vasculature (Iwamoto et al., 2014), has also been shown. Expression and tricellular junctional localization of LSR/angulin-1 at the BBB contributes to barrier maturation, as mice deficient for LSR/angulin-1 show impaired barriergenesis, which may contribute to the embryonic lethality of this mouse mutant (Mesli et al., 2004; Sohet et al., 2015).

In this study, we confirmed expression of tricellulin and LSR/angulin-1 in pMBMECs at the mRNA and protein levels; however, precise subcellular localization of tricellulin and LSR/angulin-1 using available polyclonal and monoclonal antibodies failed. High but not low inflammatory conditions induced downregulation of both tricellulin and LSR/angulin-1 (specifically isoform 2) in pMBMECs. Isoform 2 is the most highly expressed LSR/angulin-1 isoform in pMBMECs; however, the functional relevance of differential expression of the LSR/angulin-1 isoforms in establishing tricellular junctions and barrier integrity are not yet known. In accordance with observations in mouse models of stroke and MS (Sohet et al., 2015), we found that decreased expression of tricellulin and LSR/angulin-1 in IL-1β^hi^-stimulated pMBMECs was accompanied with a trend for increased permeability of pMBMEC monolayers to small molecules. Taken together, these observations suggest that both tricellulin and LSR/angulin-1, and thus tricellular junctions, play a key role in overall junctional barrier maturation of the BBB. Exactly how tricellulin and LSR/angulin-1 regulate the unique barrier stability at tricellular, and potentially bicellular, brain endothelial junctions remains to be shown, and this would require development of tools allowing the subcellular localization of tricellulin and LSR/angulin-1 to be defined.

Although we describe here, for the first time, CD4^+^ T_EM_ cell diapedesis across tricellular junctions of the BBB, immune cell extravasation through tricellular endothelial contacts in peripheral vascular beds has been observed previously. Neutrophils and monocytes have been shown to preferentially cross the tricellular contacts of cytokine-stimulated human umbilical vein endothelial cells (HUVECs) *in vitro* (Burns et al., 1997; Winger et al., 2014). Additionally, intravital and confocal microscopy has demonstrated that leukocytes cross endothelial tricellular contacts in the inflamed mouse cremaster muscle (Wang et al., 2006; Sumagin and Sarelius, 2010). Lack of expression of tricellulin and LSR/angulin-1 in peripheral vascular endothelial cells (Iwamoto et al., 2014) suggests, however, that these tricellular contacts do not form tight tricellular junctions. In fact, in contrast to the BBB, where we observed preferential CD4^+^ T_EM_ cell diapedesis via tricellular junctions under low inflammatory conditions, the diapedesis of leukocytes through tricellular endothelial contacts was observed under inflammatory conditions. Thus, we propose that CD4^+^ T_EM_ cell diapedesis across tricellular junctions of the BBB vasculature is a unique process allowing tight control of T cell entry into the CNS. Additional studies on the precise molecular structure and composition of BBB tricellular junctions will be vital to clarify the molecular underpinnings of CD4^+^ T_EM_ cell diapedesis across the BBB at these junctional sites.

In peripheral vascular beds, inflammation and increased permeability correlate with increased leukocyte extravasation via the paracellular pathway. In contrast, impaired barrier properties of the BBB correlate with increased transcellular CD4^+^ T_EM_ cell diapedesis (Abadier et al., 2015; Lutz et al., 2017; Wimmer et al., 2019). We have previously demonstrated that in IL-1β^lo^-stimulated pMBMECs, where junctional integrity is preserved, diapedesis of CD4^+^ T_EM_ cells occurs preferentially via the paracellular route. However, when junctional barrier properties are compromised, such as in IL-1β^hi^-stimulated pMBMECs, the number of CD4^+^ T_EM_ cells that migrate transcellularly increases significantly (Abadier et al., 2015). Similarly, absence of PECAM-1 in pMBMECs leads to an impairment of BBB junctional integrity, and although PECAM-1 is not required for CD4^+^ T_EM_ cell diapedesis across the BBB, its absence directs CD4^+^ T_EM_ cell diapedesis to the transcellular pathway (Wimmer et al., 2019). Altogether, it seems that modifications or loss of BBB junctional molecules results in a shift to enhanced transcellular T cell diapedesis.

Given that we observed downregulation of tricellulin and LSR/angulin-1 in IL-1β^hi^-stimulated pMBMECs, we wondered whether protein-based targeting of tricellular junctional molecules would also decrease paracellular diapedesis in favor of transcellular T cell diapedesis. Because mice that lack LSR/angulin-1 are embryonically lethal (Mesli et al., 2004), we used angubindin-1 as an LSR/angulin-1 blocking protein. Angubindin-1 has previously been shown to bind to LSR/angulin-1 in epithelia and in BBB endothelium, both *in vitro* and *in vivo*, and to remove LSR/angulin-1 from tricellular junctions (Krug et al., 2017; Zeniya et al., 2018). In our study, we observed that incubating IL-1β^lo^-stimulated pMBMECs with angubindin-1 reduced paracellular T cell diapedesis across bicellular junctions and caused a trend towards increased transcellular diapedesis, while T cell diapedesis across tricellular junctions was surprisingly not affected. Pre-treatment of IL-1β^hi^-stimulated pMBMECs with angubindin-1 did not affect the cellular pathway of T cell diapedesis. This underscores that a shift in the cellular pathway of T cell diapedesis across pMBMECs induced by angubindin-1 can only be observed under IL-1β^lo^ conditions when the junctional architecture is still intact. Of note, angubindin-1 did not induce visible changes in expression and localization of junctional or adhesion molecules and did not affect pMBMEC permeability.

In cultured epithelial cells and a rat *in vitro* model of the BBB, angubindin-1 has been found to engage the extracellular domain of LSR/angulin-1 and induce a transient relocation of LSR/angulin-1 and tricellulin from the tricellular to bicellular junctions (Krug et al., 2017). This is accompanied by a transient opening of the tricellular junctional complexes and a decrease in transepithelial and transendothelial resistance, which mimics the situation under exacerbated inflammation (Krug et al., 2017; Zeniya et al., 2018). *In vivo* application of angubinin-1 induces loss of immunodetection of LSR/angulin-1 in BBB tricellular junctions and increased BBB permeability (Zeniya et al., 2018; Krug et al., 2017).

Lack of suitable reagents prevented us from obtaining formal evidence for the localization of LSR/angulin-1 and tricellulin to tricellular junctions in pMBMEC monolayers, and thus potential angubindin-1-induced redistribution of these tricellular junctional proteins, in the present study. Fluorescently labeled angubindin-1 was observed to be rapidly taken up by pMBMECs (data not shown), suggesting internalization of LSR/angulin-1 by pMBMECs potentially also occurs outside of tricellular junctions. Because angubindin-1 can also bind to angulin-3 and partly remove LSR/angulin-1 and tricellulin from epithelial tricellular junctions (Krug et al., 2017), we cannot exclude that the effect on CD4^+^ T_EM_ cell diapedesis across pMBMECs might arise not only from the modulation of LSR/angulin-1, but also from a general effect in the function or location of tricellulin, LSR/angulin-1 and angulin-3. Thus, whether angubindin-1 disturbs tricellular and bicellular junctional compositions of pMBMECs and how its presence reduces paracellular T cell diapedesis across IL-1β^lo^-stimulated pMBMECs remains to be shown.

Targeting claudin-5 as a major component of the bicellular junctions also influenced the CD4^+^ T_EM_ cell diapedesis pathway. Similar to the effects observed upon angubindin-1 treatment, incubation of IL-1β^lo^-stimulated pMBMECs with a Cldn5 modulator decreased paracellular diapedesis through bicellular junctions, leading to increased transcellular diapedesis, with no effects seen in diapedesis through tricellular junctions. No noticeable effects on CD4^+^ T_EM_ cell diapedesis and barrier integrity were detected upon incubation of IL-1β^hi^-stimulated pMBMECs with the Cldn5 modulator. In agreement with a previous study by Neuhaus and colleagues (Neuhaus et al., 2018), no significant differences were seen in claudin-5, ZO-1, ICAM-1, VCAM-1 and VE-cadherin expression and localization. In contrast to the findings of this previous study, we did not observe an increase in permeability across pMBMEC monolayers upon incubation with the Cldn5 modulator (Neuhaus et al., 2018). This apparent difference may be due to differences in claudin-5 expression levels, tight junction assembly or architecture between the different *in vitro* BBB models used, affecting accessibility or modulation of claudin-5 by the Cldn5 modulators. In addition, in our flow chamber the Cldn5 modulator incubation was only possible from the luminal side of the pMBMEC monolayer, whereas the previous study added the cCPE proteins from the luminal and abluminal sides, which may explain the different observations (Neuhaus et al., 2018). The Cldn5 modulator most likely engages non-polymerized claudin-5 and thus sterically prevents incorporation of claudin-5 into polymeric tight junction strands. This may be achieved more efficiently if cCPE is also applied from the abluminal side (Eichner et al., 2017; Neuhaus et al., 2018). Fluorescently tagged Cldn5 modulator was observed to be internalized by pMBMECs (data not shown), suggesting that the Cldn5 modulator might interfere with junctional dynamics and/or remodeling required to establish the necessary scaffolds allowing for paracellular T cell diapedesis (Yamazaki et al., 2011). Alternatively, the Cldn5 modulator may affect the formation of endothelial-derived extracellular vesicles containing claudin-5 (Paul et al., 2016), thus shifting the cellular pathway of T cell diapedesis to transcellular sites.

Alterations of the molecular architecture and/or dynamics of BBB bicellular and tricellular BBB junctions may furthermore promote a modification in the junctional scaffold, favoring transcellular over paracellular T cell diapedesis across the BBB. A possible contribution of the endothelial cytoskeleton is likely, because tight junctions and adherens junction proteins are tightly connected to the cytoskeleton (Bauer et al., 2014). Claudin-5 is bound to the endothelial cytoskeleton through the interaction with ZO-1 and ZO-2 (also known as TJP2) (Itoh et al., 1999). Also, data from epithelial cell studies has revealed that tricellulin is involved in the regulation of F-actin organization through Tuba (also known as DNMBP), a guanine-nucleotide-exchange factor that activates Cdc42 (Oda et al., 2014). Despite the fact that the organization and composition of tricellular tight junctions of the BBB are just starting to be unveiled, we can speculate that these tricellular junctions are connected to the BBB actin cytoskeleton. Therefore, we cannot exclude the hypothesis that bicellular and tricellular junctional disarrangement in the BBB endothelium promotes cytoskeleton reorganization and thus accommodates more CD4^+^ T_EM_ cell transcellular migration events, through mechanisms yet to be clarified. On the other hand, chemotactic signaling could also be affected by altered junctions, as both transcellular and paracellular T cell diapedesis across pMBMECs depend on G-protein-coupled recetor signaling (Abadier et al., 2015; Rudolph et al., 2016). Our present study indeed suggests a specific role for BBB tricellular junctions in conveying chemotactic cues from the abluminal to the luminal side of the BBB, because presence of abluminal chemokines shifted T cell diapedesis across pMBMEC monolayers specifically to tricellular junctions.

In conclusion, by identifying BBB tricellular junctions as key structures controlling T cell diapedesis across the BBB, our study further underscores that the BBB plays an active role in controlling T cell entry into the CNS by mechanisms that are distinct from those that apply in peripheral vascular beds. Understanding the precise molecular architecture of the BBB junctional complexes and how their alteration is connected to the shift from paracellular to transcellular T cell diapedesis will allow us to improve our understanding of the role of the BBB in maintaining CNS homeostasis and immunity.

## MATERIALS AND METHODS

### Mice and mouse housing

Wild-type C57BL/6J mice were obtained from Janvier (Genest Saint Isle, France). VE-cadherin–GFP knockin C57BL/6J mice were kindly provided by Dietmar Vestweber (Max Planck Institute for Molecular Biomedicine, Muenster, Germany; Winderlich et al., 2009). Mice were housed in individually ventilated cages under specific pathogen-free conditions at 22°C and 55% relative humidity, with free access to chow and water. Animal procedures executed were approved by the Veterinary Office of the Canton Bern (permit no. BE42/14 and BE31/17) and are in line with institutional and standard protocols for the care and use of laboratory animals in Switzerland.

### *In vitro* BBB model and cell lines

Isolation and culture of primary mouse brain microvascular endothelial cells (pMBMECs) from 7–12-week-old wild-type or VE-cadherin–GFP knockin C57BL/6J mice were performed exactly as previously described (Coisne et al., 2005; Steiner et al., 2011). The unique tightness of this endothelial barrier is essential for studying the cellular migration pathway of T cells across the BBB under physiological flow, as brain endothelial cell lines not mimicking tight barrier properties do not allow delineation of different cellular pathways of T cell diapedesis across the BBB under flow *in vitro* (Steiner et al., 2010). Limitations apply to this *in vitro* BBB model, because knockdown or silencing approaches are not possible when using primary mouse brain microvascular endothelial cells that are grown to confluence over 6–7 days before being used for experiments. EpH4 cells and L cells, established and cultured as previously described (Reichmann et al., 1992; Fialka et al., 1996; Furuse et al., 1999), were grown to confluency on Matrigel-coated surfaces (Corning, NY, USA) before western blotting or immunofluorescence staining.

### T cells

For the *in vitro* live-cell imaging experiments on pMBMEC monolayers, we used the encephalitogenic CD4^+^ T_EM_ effector-memory proteolipid protein (PLP) peptide amino acids 139–153-specific T-cell line SJL.PLP7 (CD4^+^ T_EM_ cells; Engelhardt et al., 1998), as described previously (Abadier et al., 2015; Steiner et al., 2010), or *in vitro* polarized encephalitogenic T helper 1 cells (Th1 cells) from T cell receptor transgenic 2D2 mice (2D2 TCR^MOG^ mice) as previously described (Haghayegh Jahromi et al., 2019).

### *In vitro* live-cell imaging

*In vitro* live-cell imaging of CD4^+^ T_EM_ interacting with VE-cadherin–GFP knockin pMBMECs cultured in a Matrigel-coated (Corning, NY, USA) μ-Dish (35 mm, low; ibidi, Martinsried, Germany) was perfomed exactly as previously described (Abadier et al., 2015). Briefly, 10^6^ CD4^+^ T_EM_ cells/ml were perfused over a monolayer of pMBMECs in a custom-made flow chamber for the first 5 min at 0.1 dyn/cm^2^ (0.01 Pa) followed by 25 min of physiological shear at 1.5 dyn/cm^2^ (0.15 Pa). For high-resolution image acquisition over the entire pMBMEC monolayer, eight adjacent fields of view, referred to as tiles, were imaged individually with the 40× magnification objective of an inverted microscope (AxioObserver, Zeiss, Oberkochen, Germany). For evaluation purposes, tiles were combined via stitching to a larger overview using the ZEN software (blue edition, Zeiss, Oberkochen, Germany), and the number of arrested CD4^+^ T_EM_ cells on pMBMECs was counted 30 s after applying physiological flow. Diapedesis events were visualized by combining phase contrast, to properly identify T cells, with the GFP fluorescence channel, to visualize the junctions. Then, the diapedesis events were divided into three categories: transcellular, where the junctions (i.e. the GFP signal) remained intact upon diapedesis; paracellular across bicellular junctions, when the GFP signal was transiently lost at the junctions between two adjacent endothelial cells; and paracellular across tricellular junctions, when the GFP signal was transiently lost at the junctions where three endothelial cells met (exemplified in Fig. 4). Distance of T cell crawling and diapedesis duration was manually tracked for each individual T cell using FIJI software (NIH, Bethesda, MD, USA).

*In vitro* live-cell imaging of Th1 cell interaction on pMBMEC monolayers grown on nanoporous silicon nitride (NPN) membranes in the two-chamber µSiM-CVB microfluidic devices was performed as previously described (Mossu et al., 2019). Th1 cells were resuspended in migration assay medium (DMEM, 5% FBS, 4 mM L-glutamine and 25 mM HEPES) at 10^6^ cells/ml. Recombinant mouse MCP-1/CCL2 (Biolegend) and recombinant mouse RANTES/CCL5 (Biolegend) were each added at 100 ng/ml to the bottom compartment 2 h prior *in vitro* live-cell imaging. Accumulation of Th1 cells on VE-cadherin–GFP pMBMECs in the µSiM-CVB microfluidic device was allowed for 4 min at a low shear (0.1 dyn/cm^2^, 0.01 Pa), followed by physiological shear (1.5 dyn/cm^2^, 0.15 Pa) for an additional 30 min. For image acquisition, two adjacent fields of view were collected at with a 10× objective with an inverted microscope (AxioObserver, Zeiss, Feldbach, Switzerland) with phase contrast and fluorescence illumination using a monochrome charge-coupled device camera (AxioCam MRm Rev, Carl Zeiss). Image analysis was performed exactly as outlined above. A fourth category, unclear path, had to be added when analysing diapedesis events that could not be assigned to one of the three categories in the µSiM-CVB.

### Image segmentation

To determine whether the cellular pathways of T cell diapedesis across pMBMEC monolayers are random or not random, we established a baseline model corresponding to a situation where the T cells would randomly choose a location for transmigration. In such a null model, the probability of attaching to a specific location on the cell monolayer is directly proportional to the frequency of its occurrence, and therefore, based on a 2D microcopy image of the VE-cadherin–GFP pMBMEC monolayer, we measured these frequencies by counting the number of pixels belonging to each category. Boundary segmentation of endothelial junctions was perfomed using the PlantSeg algorithm (Wolny et al., 2020) originally developed for plant cell segmentation, which was found perfectly suitable for application on VE-cadherin–GFP^+^ pMBMEC monolayers. We used the ‘confocal_2D_unet_bce_dice_ds1x’ model (with GASP partitioning) for segmentation of VE-cadherin–GFP^+^ pMBMEC monolayers and validated the result by visual inspection. The input image used was a mean-intensity projection of the complete *z*-stack, from a collection of eight adjacent fields of view, as described above in the section ‘*In vitro* live-cell imaging’. This image was background corrected using the Fiji (Schindelin et al., 2012) method ‘Subtract Background’ with a radius of 20 pixels. The segmentation output was a binary image of the cell boundaries that was subsequently skeletonized and processed with skan (https://github.com/jni/skan) to identify bicellular and tricellular junctions, which were then turned into binary images as well. As bicellular and tricellular junctions are geometric lines and points without a thickness, we assigned a specific radius to them by dilating the obtained binary images. This allowed us to estimate the abundance of each category – cell surface, bicellular and tricellular junction – by counting the number of pixels belonging to each category. Overlapping regions were assigned first to tricellular junctions, then bicellular junctions and finally to the cell surface.

### Chemotaxis assays

*In vitro* polarized Th1 cells were labeled with 1 μM CellTracker Green (CMFDA Dye, Life Technologies) at 37°C with 5% CO_2_ for 30 min. Chemotactic behavior was assessed as previously described (Nishihara et al., 2020) by allowing 10^5^ Th1 cells to migrate for 2 h across laminin (from Engelbreth–Holm–Swarm murine sarcoma basement membrane, Sigma) coated Millicell filters (pore size 5.0 μm, pore density 2.0×10^6^ pores/cm^2^, growth area 0.33 cm^2^; Millicell, MCMP24H48) with 0, 1, 10, 100 or 1000 ng/ml recombinant mouse CCL2 (Biolegend) or recombinant mouse CCL5 (Biolegend) in migration assay medium (DMEM, 5% FBS, 4 mM L-glutamine and 25 mM HEPES) in the bottom compartment. Migrated Th1 cells were collected and counted with an Attune NxT Flow Cytometer (Thermo Fisher Scientific) by gating on CMFDA-positive live cells.

### Serial block-face scanning electron microscopy

CD4^+^ T_EM_ cells were allowed to interact with pMBMECs under physiological flow conditions (1.5 dyn/cm^2^, 0.15 Pa) for 13 min, a timepoint at which most of the T cells were undergoing diapedesis. Afterwards, the assay was fixed by perfusion of 2.5% glutaraldehyde, 2 mM CaCl and 0.15 M cacodylate (pH 7.4) through the flow chamber. Samples were rinsed three times for 5 min in ice-cold 0.15 M Na cacodylate and then incubated in 0.15 M Na cacodylate solution containing 2% OsO_4_ and 1.5% potassium ferrocyanide for 1 h at room temperature. After a washing step with water, samples were incubated in 0.64 M pyrogallol for 20 min at room temperature, then were washed again. The samples were incubated in 2% OsO_4_ for 30 min at room temperature, and after another washing step, incubated overnight in a solution of 0.15 M gadolinium acetate (LFG Distribution, Lyon, France) and 0.15 M samarium acetate (LFG Distribution, Lyon, France). The next day, samples were washed and incubated in Walton's lead aspartate (Walton, 1979) at 60°C for 30 min, then rinsed with water. After staining, the samples were dehydrated in a graded ethanol series (20%, 50%, 70%, 90%, 100%, 100%) at 4°C, each step lasting 5 min. They were then embedded with Durcupan resin mixed with ethanol at ratios of 1:3 (v/v), 1:1 and 3:1, each step lasting 2 h. Samples were then infiltrated with pure Durcupan overnight. The samples were transferred to fresh Durcupan, and the resin was polymerized for 3 days at 60°C. Sample blocks were mounted on aluminum pins (Gatan, Pleasonton, CA, USA) with a conductive epoxy glue (CW2400, Circuitworks, Kennesaw, GA, USA). Care was taken to have osmicated material directly exposed at the block surface in contact with the glue in order to reduce specimen charging under the electron beam. Pyramids with a surface of ∼500×500 μm^2^ were trimmed with a razor blade and imaged with an FEI Quanta 250 FEG with Gatan 3View2XP. A total of 2000 images were acquired per sample, with a slice thickness of 60 nm and a pixel size of 0.012 μm. For the visualization of the images and full analysis of the dataset, we used 3dmod software (University of Colorado, Boulder, CO, USA) and FIJI (NIH, Bethesda, MD, USA). Manual segmentation was performed using FIJI (NIH, Bethesda, MD, USA) and UCSF Chimera (University of California, San Francisco, CA, USA).

### Analysis of T cell protrusions

For the analysis of the images taken by SBF-SEM, we used the program 3dmod (University of Colorado Boulder, Colorado, USA). For each condition (IL-1β^low^- or IL-1β^high^-stimulated pMBMECs), ten T cells that were in the stage of probing were randomly chosen to be analyzed. We defined protrusions as T cell processes interfering with the endothelium. We determined the range from the smallest protrusion observed, having a diameter (in the *x*-axis) of roughly 90 nm and a depth (in the *y*-axis) of roughly 40 nm, to the biggest protrusion observed, having a diameter (in the *x*-axis) of roughly 1700 nm and a depth (in the *y*-axis) of roughly 800 nm.

### *In vitro* immunofluorescence staining

Confluent pMBMEC monolayers were stained precisely as previously described (Lazarevic and Engelhardt, 2016). Briefly, pMBMECs were gently washed with Dulbecco's phosphate buffered saline (DPBS) and subsequently fixed with either 1% paraformaldehyde (PFA; MERCK, Darmstadt, Germany) in DPBS at room temperature for 10 min or with −20°C methanol (for claudin and occludin staining), for 1 min. Unspecific binding was blocked by incubating the cells with Tris-buffered saline (TBS) containing 5% skim milk and 0.2% Triton X-100 (BioRad Laboratories, Hercules, CA, USA). Primary and secondary antibodies were incubated for 1 h at room temperature, and the staining was analyzed using a Nikon Eclipse E600 microscope connected to a Nikon Digital Camera DXM1200F, with the Nikon NIS-Elements BR3.10 software (Nikon, Egg, Switzerland). Images were processed and mounted using Adobe Illustrator software.

### *In vitro* permeability assays

*In vitro* permeability of the pMBMEC monolayers was assessed by measuring the clearance of Alexa Fluor 680-labeled 3 kDa dextran (Thermo Fisher Scientific, Carlsbad, CA, USA) and of 0.45 kDa Lucifer Yellow (Sigma-Aldrich Chemie GmbH, Buchs, Switzerland), exactly as described previously (Cecchelli et al., 2014, 1999; Coisne et al., 2005; Steiner et al., 2010). In brief, the fluorescent tracers diffusing across the pMBMEC monolayers were collected from the bottom well every 20 min for a total of 60 min. Fluorescence intensity for Alexa Fluor 680-labeled 3 kDa dextran and Lucifer Yellow was measured by infrared imaging (Odyssey Quantitative Fluorescence Imaging System, LI-COR, Bad Homburg, Germany) and with a Tecan Infinite M1000 multi-well reader (Tecan Trading AG), respectively. The endothelial permeability coefficient (Pe) was calculated using the clearance principle to obtain a concentration-independent transport parameter, as previously described in detail (Coisne et al., 2005). The experiments were performed in triplicates for each condition.

### RT-qPCR analysis

RNA extraction from pMBMEC culture was done using the High Pure RNA Isolation Kit (Hoffman-La Roche, Basel, Switzerland). Afterwards, cDNA was obtained from the total RNA of each sample with the SuperScript III First-Strand Synthesis System (Invitrogen, Carlsbad, CA, USA), and RT-qPCR was done as previously described (Lyck et al., 2009). Selected sets of genes to be analyzed were tested using Takyon Low Rox SYBR MasterMix dTTP Blue (Eurogentec, Liège, Belgium). The sequences of the primers used were as follows: for tricellulin, 5′-CTGGCCTGACCGAGACAA-3′ and 5′-CAACGACGGGTCATTTATCC-3′; for LSR/angulin-1, 5′-GCTGTGACCCTGGGAGACTA-3′ and 5′-CGAAGGTCAGGTCAGCATTT-3′; for angulin-2, 5′-AATGTGGAGAGGCGCTTG-3′ and 5′-TGTATGATCCAAGAAGCAGTATGG-3′; and for angulin-3, 5′-CAGTTGCTGCTGCTATGTCC-3′ and 5′-TGCTTTCCCTGCTTCATACA-3′. β-actin was used as the endogenous control, with the primer sequences of 5′-AAGGCCAACCGTGAAAAGAT-3′ and 5′-GTGGTACGACCAGAGGCATAC-3′. All RT-qPCR reactions were done in triplicates for each sample and performed using the Viia7 Real-Time PCR System (Thermo Fisher Scientific, Waltham, MA, USA). ΔCT value was obtained (average CT value of target gene−average CT value of β-actin).

### Antibodies and cytokines

The primary and secondary antibodies used in this study are described in Table S1. IL-1β (PeproTech, Rocky Hill, NJ, USA) stimulation of pMBMEC monolayers was done for 16–20 h at a concentration of 0.05 ng/ml (IL-1 β^lo^) or 20 ng/ml (IL-1 β^hi^). Angubindin-1, used to detect LSR/angulin-1, was coupled with DyLight 550 NHS ester with the DyLight Antibody labeling kit (Thermo Fisher Scientific, Carlsbad, CA, USA), according to the manufacturer's instructions.

### SDS–PAGE

pMBMECs from wild-type C57BL/6J mice, EpH4 and L-cells were lysed in RIPA buffer [10 mM Tris-HCl (pH 8.0), 1 mM EDTA solution, 1% Triton X-100, 0.1% sodium deoxycholate, 0.1% SDS, 140 mM NaCl and 1 mM PMSF], in the presence of protease inhibitor cOmplete ULTRA Tablets, Mini, EDTA-free, EASYpack (1 tablet/10 ml buffer; Roche Diagnostics, Mannheim, Germany). Protein concentration was measured using the Pierce BCA Protein Assay Kit (Thermo Scientific Pierce Protein Biology, Waltham, USA), according to the manufacturer's instructions. A total of 5–20 μg of each sample was loaded onto a 10% SDS-polyacrylamide gel and transferred to a nitrocellulose membrane (Amersham Protan, GE Healthcare, United Kingdom) using a Trans-Blot Turbo transfer system (BioRad Laboratories, Hercules, CA, USA), according to the manufacturer's instructions. Membranes were blocked with Rockland Buffer (Rockland, Limerick, PA, USA) for 1 h at room temperature and incubated overnight at 4°C with the primary antibodies (Table S1). On the following day, membranes were washed and incubated with secondary antibodies (Table S1), for 1 h at room temperature. Proteins were detected using an Odyssey near infrared imaging system and software (LI-COR Biotechnology, Lincoln, NE, USA). Band intensity was quantified using FIJI software (NIH, Bethesda, MD, USA) and normalized against β-actin intensity.

### Blocking proteins and blocking assays

To target claudin-5 as a component of endothelial bicellular junctions, the following recombinant proteins were used: GST–cCPE_Y306W/S313H (referred to in this study as ‘claudin-1, -3, -4, -5 modulator’) and GST–cCPE_N218Q/Y306W/S313H (referred to in this study as ‘claudin-5 modulator’). GST–cCPE_Y306/L315A (referred to in this study as ‘control protein’) was used as a negative control. The proteins were produced as described previously (Neuhaus et al., 2018; Protze et al., 2015). The pMBMEC monolayers were incubated with these modulators at a concentration of 10 µg/ml for 16 h, and the modulators were removed with a washing step immediately before performing the immunofluorescence staining, *in vitro* permeability assays or *in vitro* live-cell imaging experiments.

To target LSR/angulin-1 at the tricellular junctions, angubindin-1 (Ib421-664) was used. C2II592-721 (referred to in this study as ‘control protein’) was used as a negative control. Both proteins were produced as previously described (Krug et al., 2017; Nagahama et al., 2004; Blocker et al., 2000). Both proteins were incubated with the pMBMEC monolayer at a concentration of 5 µg/ml for 16 h and removed with a washing step immediately before performing the immunofluorescence staining, *in vitro* permeability assays or *in vitro* live-cell imaging experiments.

### Statistics

Statistical analysis was performed using GraphPad Prism 6.0 software (San Diego, CA, USA). To compare two groups, an unpaired *t*-test with Welch's correction was performed and for the comparison of three groups, we performed a one-way ANOVA with post hoc Tukey test. A *P*-value <0.05 was used as the level of significance.

## Supplementary Material

Supplementary information

Reviewer comments
