# Supplementary material for: Brain endothelial tricellular junctions as novel sites for T cell diapedesis across the blood–brain barrier
Source: J Cell Sci. 2021 Apr 26;134(8):jcs253880. doi: 10.1242/jcs.253880 (PMC8121105; doi:10.1242/jcs.253880)
Supplement: Supplementary information [file joces-134-253880-s1.pdf]

Table S1 – List of the antibodies used in this study

Primary antibodies						
**Antigen**	**Company**	**Catalog no.**	**Lot no.**	**Host species**	**Concentration**	
					**Western blot**	**IF staining tissue culture**
Mouse Claudin-5	Thermo Fisher Scientific (Invitrogen)	34-1600	1105603A	Rabbit polyclonal IgG	1.25 µg/mL	1.25 µg/mL
Mouse Occludin	Thermo Fisher Scientific (Invitrogen)	71-1500	RG235307	Rabbit polyclonal IgG	1.25 µg/mL	1.25 µg/mL
Mouse β-actin	Merck	A5316	052M4793	Mouse monoclonal IgG2a	1:3000	n.d.
Mouse ZO-1	Thermo Fisher Scientific (Invitrogen)	61-7300	QG215368	Rabbit polyclonal IgG	n.d.	1.25 µg/mL
Mouse MARVELD2/ Tricellulin	Thermo Fisher Scientific (Invitrogen)	48-8400	SH258222	Rabbit polyclonal IgG	0.25 µg/mL	2 µg/mL
Mouse MARVELD2/ Tricellulin	in house Furuse lab[&]	n.a.	n.a.	Rat monoclonal IgG	n.d.	supernatant undiluted
Mouse LSR/angulin-1	Merck	HPA007270	I106879	Rabbit polyclonal IgG	1 µg/mL	n.d.
Mouse LSR/angulin-1	In house Furuse lab[&]	n.a.	n.a.	Rat monoclonal IgG	n.d.	supernatant undiluted
Mouse VCAM-1	In house	Clone 9DB3	n.a.	Monoclonal rat IgG2a	n.d.	20 µg/mL
Mouse ICAM-1	In house	Clone 25ZC7	n.a.	Monoclonal rat IgG2a	n.d.	20 µg/mL
Mouse ICAM-1	Custom made, Eurogentec	n.a.	n.a.	Rabbit polyclonal IgG	1:1000	n.d.
F-actin TRITC-Phalloidin	Merck, Switzerland	P1951		Amanita phalloides	n.d.	1:200
**Secondary antibodies**						
**Antigen**	**Company**	**Catalog no.**	**Lot no.**	**Antibody/ reagent**	**Concentration**	
					**Western blot**	**IF staining tissue culture**
Rabbit IgG (H+L)	Thermo Fisher Scientific (Invitrogen)	A21109	1716991	Goat polyclonal IgG Alexa Fluor® 680	1:10000	n.d.
Mouse IgG (H+L)	Rockland Immunochemicals	605-732-125	17583	Goat polyclonal IgG IRDye® TM 800	1:10000	n.d.
Rat IgG (H+L)	Jackson ImmunoResearch	112-165-003	2338240	Goat polyclonal IgG Cy3	n.d.	1:200
Rabbit IgG (H+L)	Thermo Fisher Scientific (Invitrogen)	A27034	1214851	Goat polyclonal IgG Alexa Fluor® 488	n.d.	1:200

*IF – immunofluorescence; $ n.d. – not done; § n.a. – not applicable; & (Iwamoto et al., 2014)

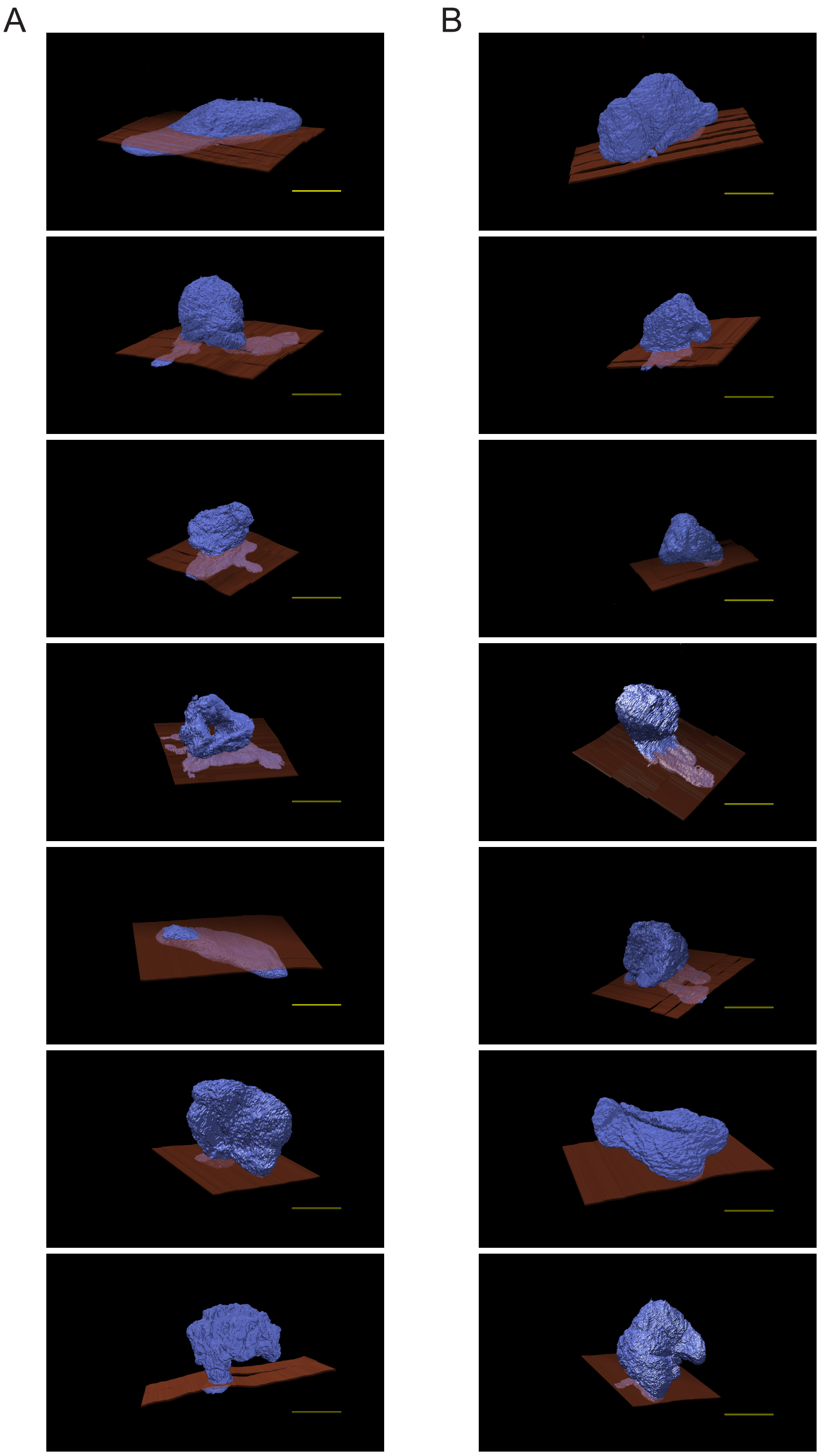

Figure S1 - 3D segmentation of the morphology of 14 T-cell nuclei, interacting with either IL-1β^{lo} or IL-1β^{hi} stimulated pMBMECs (A and B, respectively). Scale bar = 5 µm

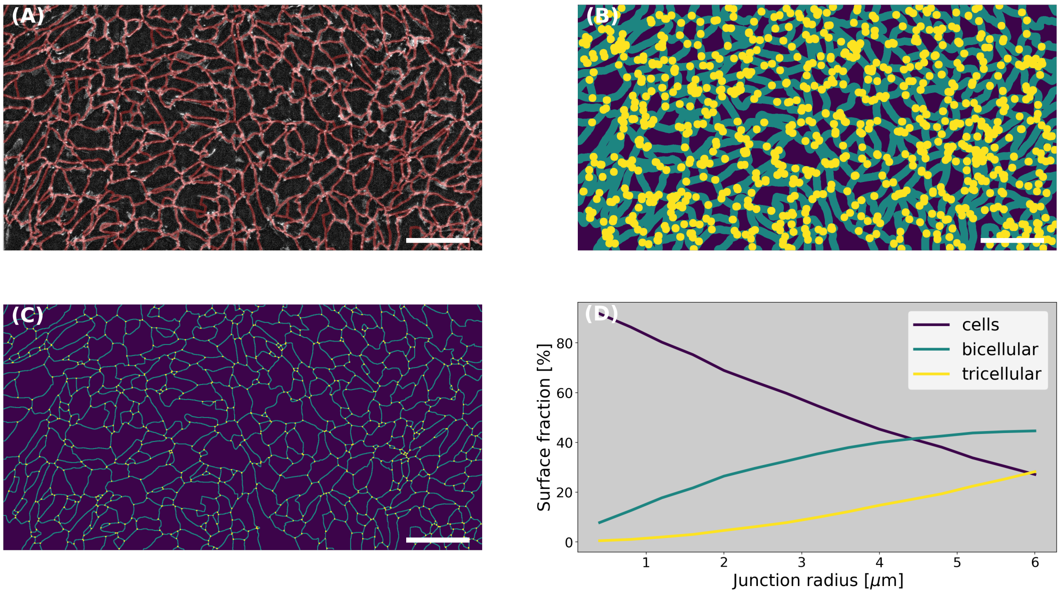

Figure S2 – **Radomization of the cellular pathway of T-cell diapedesis across pMBMEC monolayers** **(A)** Boundary segmentation (red lines) of a VE-cadherin-GFP pMBMEC monolayer is shown. Underlying GFP^+ pMBMEC junctions are visible in grey. **(B,C)** Maps assuming a 6 μm (B) or 1 μm (C) radius for the junctions. Paracellular junctions shown in green, tricellular junctions in yellow. **(D)** shows the fraction of pixels belonging to a given category as a function of the junction radius. With increasing radius, the surface fraction of the junctions becomes larger while the fraction of pixels for the cell surface becomes smaller. Although this model does not allow to estimate the true thickness of junctions, it allows to determine that only under the assumption of very large radii (> 4 μm) cellular junctions become dominant in the null model. Scale bar = 100μm.

Journal of Cell Science • Supplementary information

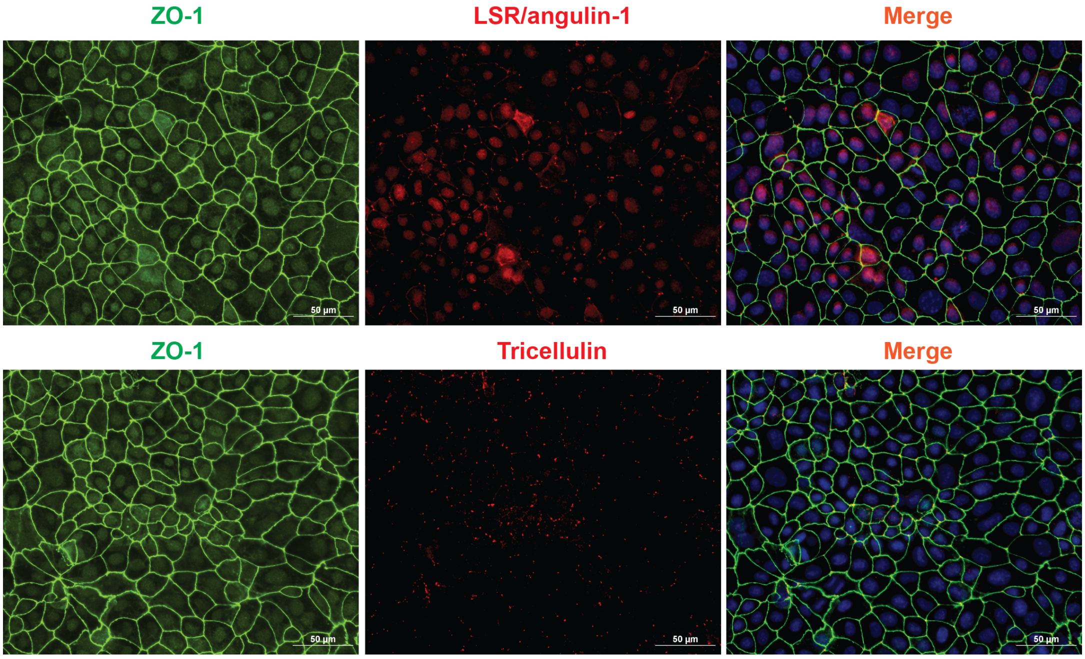

Figure S3 – Detection of tricellulin and LSR/angulin-1 in epithelial monolayers
Immunofluorescence staining of ZO-1 (green) and tricellulin and LSR/angulin-1 (red) in EpH4 monolayers. Scale bar = 50µm.

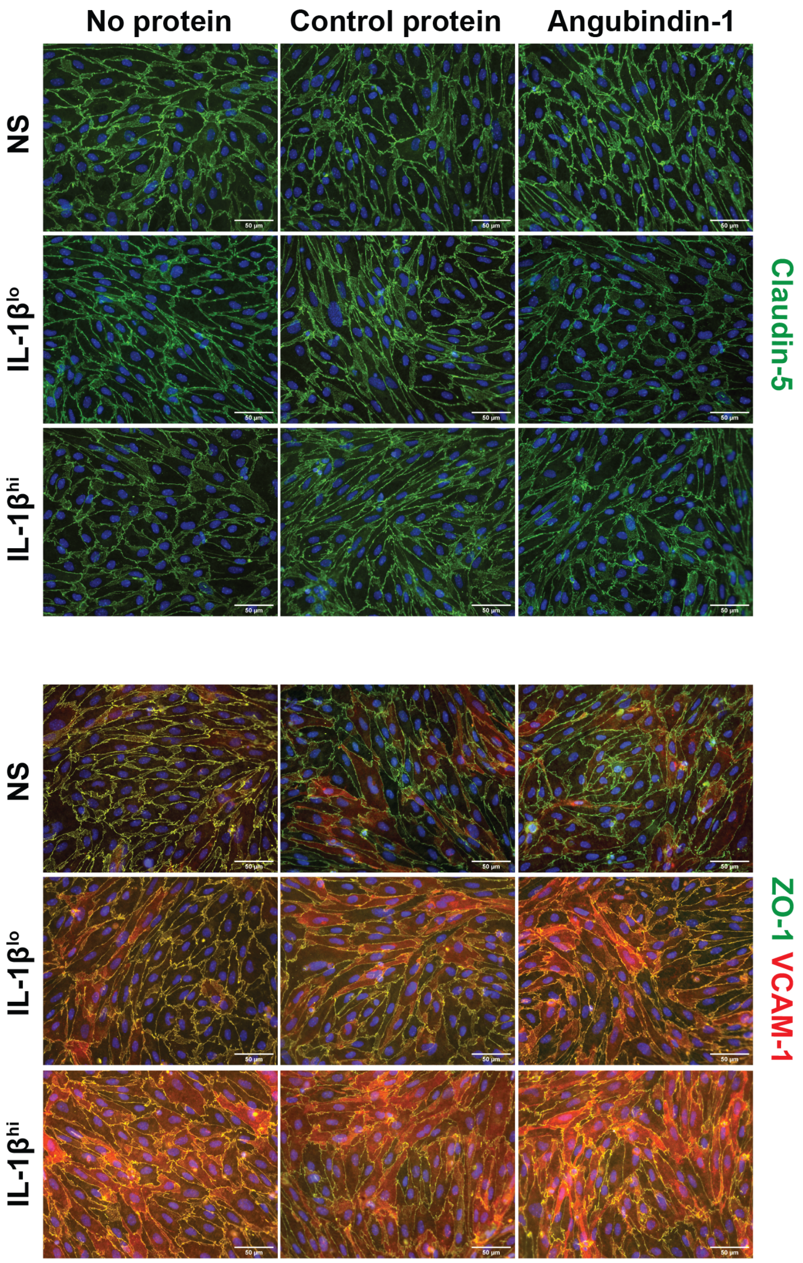

Figure S4 – Protein-targeting of angulin-1 reduces paracellular T-cell diapedesis

Immunofluorescence staining of claudin-5 and ZO-1 (green) and VCAM-1 (red) in pMBMEC monolayers. Comparable staining was obtained for these proteins, in either unstimulated, IL-1β^{lo} or IL-1β^{hi} stimulated pMBMECs, in the presence or absence of absence of angubindin-1. Nuclei were stained with DAPI in blue. Three independent experiments were performed. Scale bar = 50µm.

Journal of Cell Science • Supplementary information

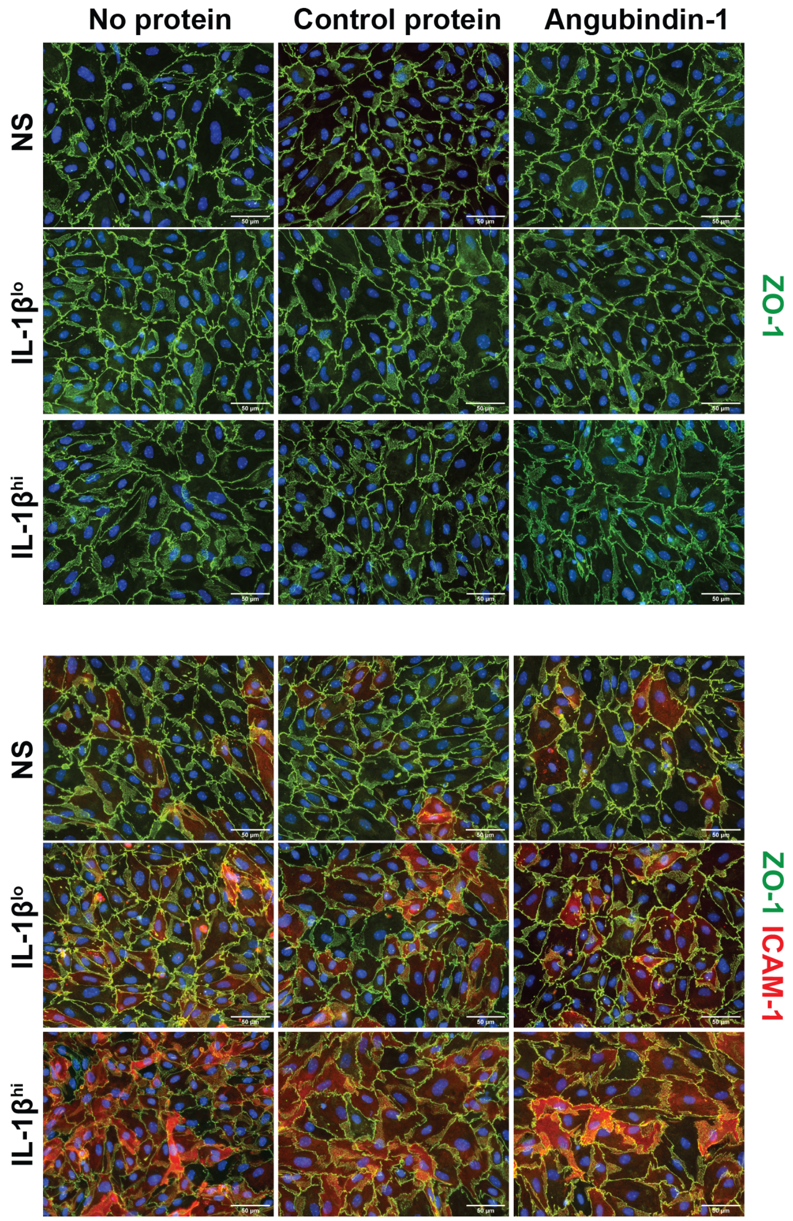

Figure S5 - Protein-targeting of angulin-1 reduces paracellular T-cell diapedesis
Immunofluorescence staining of ZO-1 (green) and ICAM-1 (red) in pMBMEC monolayers. Comparable staining was obtained for these proteins, in either unstimulated, IL-1β^{lo} or IL-1β^{hi} stimulated pMBMECs, in the presence or absence of angubindin-1. Nuclei are stained with DAPI in blue. Three independent experiments were performed. Scale bar = 50µm.

Journal of Cell Science • Supplementary information

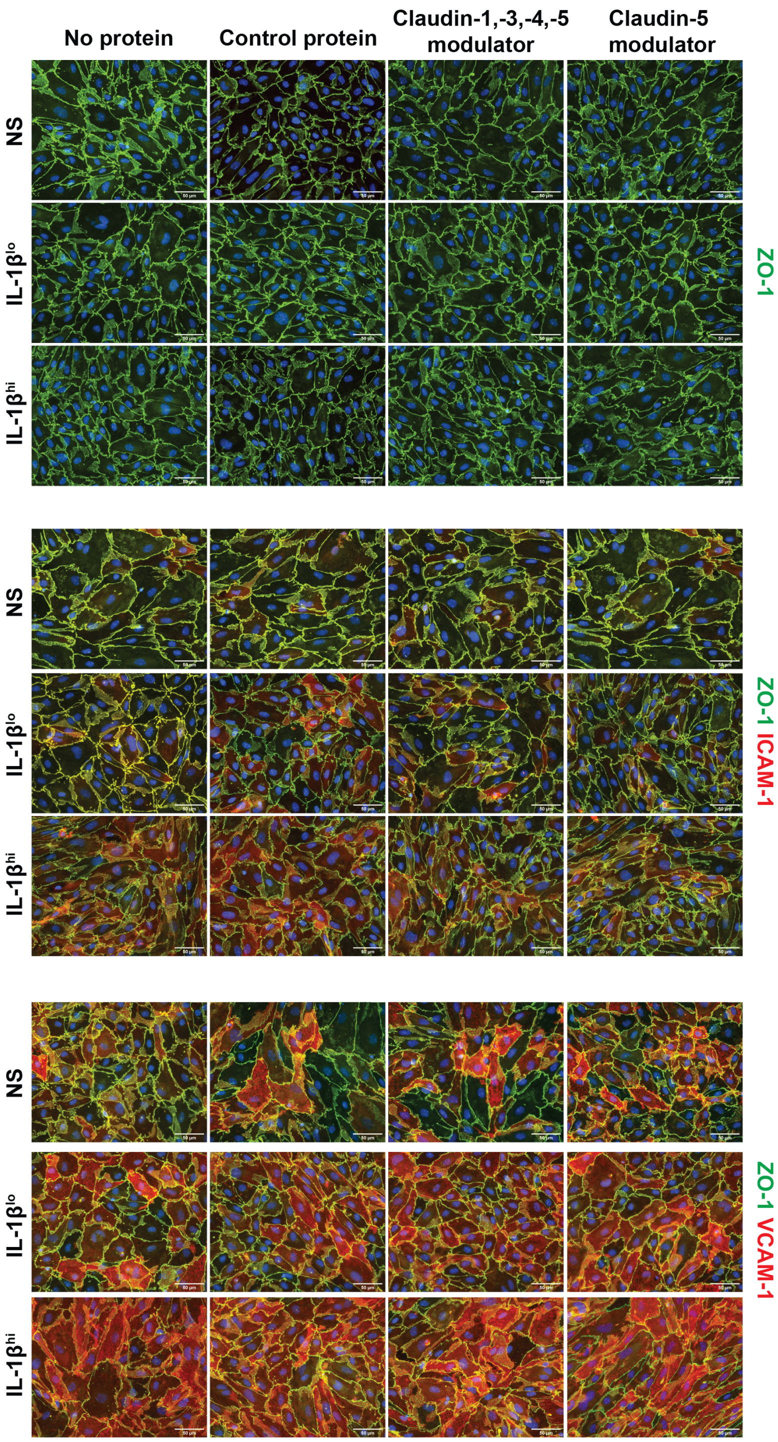

Figure S6 - Targeting endothelial claudin-5 decreases paracellular T-cell diapedesis across bicellular junctions

Immunofluorescence staining of ZO-1 (green) and ICAM-1 and VCAM-1 (red) in pMBMEC monolayers. Comparable staining was obtained for these proteins, in either non stimulated (NS), IL-1β^{lo} or IL-1β^{hi} stimulated pMBMECs, in the presence or absence of the cCPE proteins. Nuclei were stained with DAPI in blue. Three independent experiments were performed. Scale bar = 50µm.

Journal of Cell Science • Supplementary information

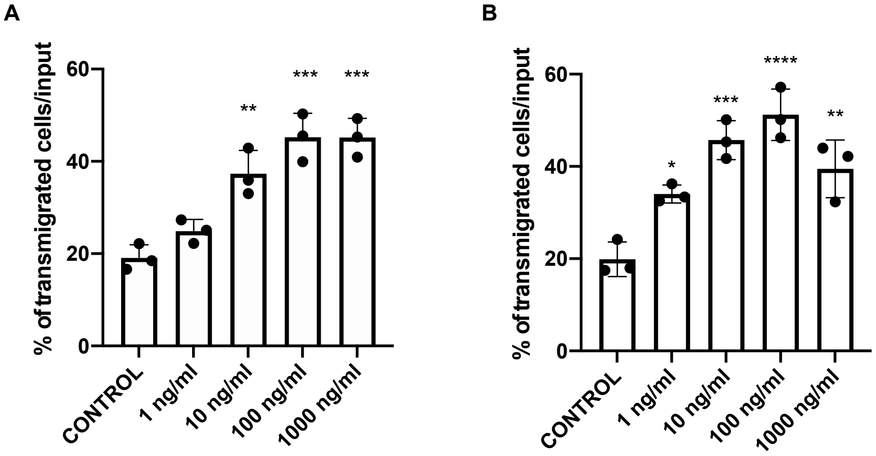

Figure S7- Th1 cells chemotaxis towards CCL2 and CCL5.

The percentage of Th1 cells that have migrated over 2 hours across laminin coated 5 µm pore size Millicell filters towards 0 (CONTROL), 1, 10, 100, 1000 ng/mL recombinant mouse CCL2 (a) and recombinant mouse CCL5 (b) in the bottom compartment. Statistical analysis: one-way ANOVA followed by Tukey's multiple comparison test ($p < 0.05$ = *, $p < 0.01$ = **, $p < 0.001$ = ***, $p < 0.0001$ = ****). Each figure shows mean + SD of one representative experiment done in triplicates.

Journal of Cell Science • Supplementary information
