# Supplementary material for: Brain endothelial tricellular junctions as novel sites for T cell diapedesis across the blood–brain barrier
Source: J Cell Sci. 2021 Apr 26;134(8):jcs253880. doi: 10.1242/jcs.253880 (PMC8121105; doi:10.1242/jcs.253880)
Supplement: Reviewer comments [file joces-134-253880_review_history.pdf]

Journal of Cell Science

Brain endothelial tricellular junctions as novel sites for T cell diapedesis across the blood-brain barrier

Mariana Castro Dias, Adolfo Odriozola Quesada, Sasha Soldati, Fabio Bösch, Isabelle Gruber, Tobias Hildbrand, Derya Sönmez, Tejas Khire, Guillaume Witz, James L. McGrath, Joerg Piontek, Masuo Kondoh, Urban Deutsch, Benoit Zuber and Britta Engelhardt
DOI: 10.1242/jcs.253880

Editor: Daniel Billadeau

Review timeline

Original submission:	3 September 2020
Editorial decision:	13 October 2020
First revision received:	17 February 2021
Editorial decision:	8 March 2021
Second revision received:	9 March 2021
Accepted:	12 March 2021

Original submission

First decision letter

MS ID#: JOCES/2020/253880

MS TITLE: Brain endothelial tricellular junctions as novel sites for T-cell diapedesis across the blood-brain barrier

AUTHORS: Mariana Castro Dias, Adolfo Odriozola Quesada, Isabelle Gruber, Tobias Hildbrand, Derya Soenmez, Joerg Piontek, Masuo Kondoh, Urban Deutsch, Benoit Zuber, and Britta Engelhardt
ARTICLE TYPE: Research Article

We have now reached a decision on the above manuscript.

To see the reviewers' reports and a copy of this decision letter, please go to: https://submit-jcs.biologists.org and click on the 'Manuscripts with Decisions' queue in the Author Area. (Corresponding author only has access to reviews.)

As you will see, the reviewers raise a number of substantial criticisms that prevent me from accepting the paper at this stage. It is clear that several experiments will need to be performed in order to support the claims made in the manuscript. They suggest, however, that a revised version might prove acceptable, if you can address their concerns. If you think that you can deal satisfactorily with the criticisms on revision, I would be pleased to see a revised manuscript. We would then return it to the reviewers.

We are aware that you may be experiencing disruption to the normal running of your lab that makes experimental revisions challenging. If it would be helpful, we encourage you to contact us to discuss your revision in greater detail. Please send us a point-by-point response indicating where you are able to address concerns raised (either experimentally or by changes to the text) and where you will not be able to do so within the normal timeframe of a revision. We will then provide further guidance. Please also note that we are happy to extend revision timeframes as necessary.

Please ensure that you clearly highlight all changes made in the revised manuscript. Please avoid using 'Tracked changes' in Word files as these are lost in PDF conversion.

I should be grateful if you would also provide a point-by-point response detailing how you have dealt with the points raised by the reviewers in the 'Response to Reviewers' box. Please attend to all of the reviewers' comments. If you do not agree with any of their criticisms or suggestions please explain clearly why this is so.

Reviewer 1

Advance summary and potential significance to field

The authors report on the role of endothelial tricellular junctions as novel sites for T-cell diapedesis, specifically at brain endothelial cell regions.
Although the idea of preference for leukocytes to take tricellular junctions is not new per se, it has never been shown for T-cells crossing brain EC. And as this work is done thoroughly, it is a valuable addition to the information that is out there to the field.

Comments for the author

The authors show that T-cells prefer to cross tricellular junctions and depend on the presence of certain proteins that "build" tricellular junctions. If such proteins are absent, T-cells manage to go through transcellularly. This also indicates that blocking junction route may not be an efficient target for treatment.

The work is done thoroughly, and the figures are clear presented and conclusions drawn from the results are sound. However, at the end, it is not clear how they target tricellular junctions. I miss the proof that they actually effectively targeted tricellular junctions.

I have some comments and potential improvements that I have listed below.

1. In fig 1, ICAM-1 and VCAM-1 expression is shown. At higher concentrations IL1, it is clear that the expression shows a heterogenic expression pattern. The authors should at least mention this in the text.
2. I would recommend including some arrows n figure 2, in particular when talking about certain cellular structures or organelles. Indicate the cell-cell junctions better for example.
3. I would suggest changing the abbreviation for Angulin: Ang-1, Ang-2.
Ang reminds me too much to Angiopoietin….
4. The effect on tricellular junction proteins is of interest. However, no clear functionality can be directed to it. The approach by using angubindin-1 did not really gave any satisfactory answers. Also no increase in ICAM-1, VCAM-1 etc. And yet, the authors show an increase in adhesion of T-cells (figure 6C). I am also not sure why the authors continued with this approach? What is angubindin-1 supposed to do? It did not affect permeability. Why not silencing tricellulin or overexpressing it to study its functionality better? I think that the authors should try to improve the latter part of the Ms, in particular the role of the tricellular junctions on T-cell TEM.
5. And in the last paragraph of the result section they state that: "To address if modulation of tricellular junctions would affect diapedesis…." But did the authors actually modulate tricellular junctions, other than IL1 treatment??
6. The last statement: "…targeting tricellular junctional components in brain endothelium favors transcellular T-cell diapedesis… ", I am not convinced that they have actively targeted tricellular junctions. For this, I would suggest at least a knock down.
7.

Minor:
- ypo in abstract: junctional
- half way page 5: word missing: stimulated ECs

Reviewer 2

Advance summary and potential significance to field

Dias et al study diapedesis of T cells across cerebral endothelial monolayers under flow, a model which has consistently and in various labs delivered important insights into the molecular details of transendothelial leukocyte migration in general and key mechanistic differences between different molecular beds. Here the authors have studied diapedesis primarily by time lapse microscopy and crucially, by painstaking reconstructive serial block face scanning electron microscopy. They found that diapedesis location occurred paracellularly, both at bicellular and tricellular endothelial contact sites, as well as transcellularly. Additional data point to the importance of tricellular junction composition in determining the diapedesis route. Overall, it is at this point that this study falls short and does not deliver unequivocal data to support potentially important conclusions drawn.

If the authors can fix that, this study will make an important contribution to how the BBB regulates immune cell infiltration, in particular how it is dependent on the presence of specialised tricellular junction proteins of the MARVEL family.

Comments for the author

Major comments:

The model of IL-1b(low) and (hi) is not new and is mostly well characterised. However, in the abstract and the discussion, it is claimed that results from the IL-1b(low) cells are representative of cerebral immune cell under conditions of immune surveillance. What is the basis for likening this in vitro cell system to cerebral immune surveillance (for which the role of the microvasculature, to the best of my knowledge, is still not unequivocally demonstrated)?

Figure 1A: This figure is claimed to show unaltered TJ distribution. However, in IL1b treated cells TJ continuity appears impaired. In addition, judging by the nuclear stain, some of the IL1b-stimulated monolayers appear to consist of piled up endothelial cells.

Robustness of permeability measurements:
The authors have shown in a previous publication (Abadier et al) that barrier properties are altered in response to IL1b pre-treatment. In the present dataset, there is a trend, which is not borne out statistically. I propose that the authors alter their method to reduce the assay error or increase the number of independent repeats to unequivocally establish if these trends are real. While this is not so much a problem in figure 1, it is much more so for statements on barrier integrity following intervention with angubindin or the claudin modulators (Figure 6).
In the discussion the authors state that they "found that decreased expression of tricellulin and LSR/angulin in IL-1b9hi) stimulated pMBMECs was increased permeability to small molecules". Where is statistically robust data illustrating this shown?
Permeability (flux) measurements were carried out using cells cultured in transwells in the absence of flow. Whilst these are conditions considerably different to those used for the transmigration assays, I do not consider this a problem per se. However, in the discussion the authors claim that the reason for not reproducing published effects of CLDN-5 modulation (Neuhaus et al) is due to their system not allowing access to the basal side of the ECs, which clearly cannot be true.

EM and time lapse imagery:
Figure 2: The authors have made a great effort to gather EM data on transmigrating T cells. However, more illustrating details should be shown so that the amount of ultrastructural detail can be fully appreciated. For instance, more arrowing of key features and colour shading of the two cell types is often used for similar datasets.
Additionally, the authors use these images to derive quantitative data on paracellular and transcellular transmigration events. None of the images shown in this figure allow such a categorisation and images of representative examples must be shown.
Figure 3. Panel A: Again, additional colour shading of the T and EC cell would be of great help to fully appreciate the images. Additionally, what is the proof that the T cell purported to represent a paracellular transmigration event is not ultimately diapedesing through the nearby junction? Given that these EM pictures only show isolated time points, it is necessary that the authors demonstrate that the cells shown fulfil the criteria of "at least part of their nucleus inserted through the pMBMEC monolayer".
Panel B: The remainder of the manuscript relies on diapedesis differences across IL-1b(low) and (high) cells.
Some kind of statistical analysis must be undertaken to demonstrate that this does not represent a random segregation of observations.
Figure 4: There are several problems with the imaging data shown here. What is the line separating different parts of exposure in the VEcad images? Without outlining the position and circumference of the T cells in the VEcad images, it is not possible to fully determine where exactly diapedesis occurs.

There are many instances where the authors fail to reproduce important prior observations in their experimental cells system and this impacts the overall value of the study (see also above comment on barrier measurement):
Other studies have neatly shown the localisation of MARVEL proteins to tricellular junctions in cultured endothelial cells or intact blood vessels. Thus it is insufficient for the authors to simply state that they have not been able to achieve staining for tricellulin or angulins, in particular since the reduced level of expression of these proteins as measured by WB is mild and may well be due to their complete disappearance at a small subset of tricellular contact points (which ultimately no longer serve as diapedesis sites). Given that the quantitative differences the authors base their conclusions on, are subtle, a more in-depth analysis of MARVEL protein localisation with respect to diapedisis routes is

required. An even more damning interpretation of the data shown could be that, whilst these MARVEL proteins are expressed, they fail to localise appropriately in these mouse endothelial cell cultures, which would invalidate most of the conclusions drawn.
The authors claim that expression levels of MARVEL proteins are important for the location of diapedesis. The argument for using proteins/peptides interfering with their function is justified by the lethality of ko mice. Why have MARVEL protein expression not been modulated using siRNA (which deliver a measurable biochemical endpoint demonstrating successful intervention)?
Overall the protein/peptide tools used to modulate MARVEL protein functions have been well characterised in other, published datasets but not here. The authors themselves concede that e.g. CLDN interference may depend on access to the basal side of the endothelium. Proof of successful intervention must be provided.

Overall, the manuscript is long-winded with too many sections inserted as important arguments in the absence of clear data. The authors should stick to the main message and produce a clear, linear narrative to convey the message.

The authors use many very definitive adjectives to describe their findings and emphasise their impact. However, these should be adapted to reflect findings more realistically. 'Novel' is used for the observation of migration at tricellular junctions. However, as covered in detail in the discussion, this phenomenon has been described before. I understand that the authors believe that their data show clear differences between diapedesis at the BBB and the periphery, however, to reach this conclusion more molecular detail must be shown (as discussed above).
Diapedesis at tricellular junctions is also noted as the 'preferred route', however it makes up for less than 50% of all migration events, and in the absence of any meaningful statistical evaluation may be similar to migration at bicellular junctions.

Minor:
In the discussion the authors describe tricellulin as a MARVEL family protein, however angulins are also part of this family.

Whilst very interesting, the reconstruction work illustrating nuclear deformation in T cells during diapedesis does not serve any purpose with regard to the message of this manuscript.

First revision

Author response to reviewers' comments

UNIVERSITÄT
BERN

To
Dr. Daniel Billadeau
Editor
Journal of Cell Science

Medizinische Fakultät
Theodor Kocher Institut
Prof. Dr. Britta Engelhardt
Direktorin

Bern, 13.02.21

Submission of our revised manuscript JOCES/2020/253880 entitled "Brain endothelial tricellular junctions as novel sites for T-cell diapedesis across the blood-brain barrier» for publication in the *Journal of Cell Science*

Dear Dr. Billadeau

Please find enclosed our revised manuscript entitled "Brain endothelial tricellular junctions as novel sites for T-cell diapedesis across the blood-brain barrier" for publication in the *Journal of Cell Science*.

We thank the Reviewers for their very insightful comments to our manuscript which have initiated additional analyses and experiments and significantly improved our manuscript. We have performed additional experiments as requested and revised the manuscript according to the queries of the Reviewers. Please find our point-by-point reply below. Changes in the revised manuscript are highlighted in blue.

Reviewer 1 Advance Summary and Potential Significance to Field:
The authors report on the role of endothelial tricellular junctions as novel sites for T-cell diapedesis, specifically at brain endothelial cell regions. Although the idea of preference for leukocytes to take tricellular junctions is not new per se, it has never been shown for T-cells crossing brain EC. And as this work is done thoroughly, it is a valuable addition to the information that is out there to the field.
Comment: We thank the reviewer for this positive statement. We agree that leukocyte migration across tri-cellular endothelial junctions has been observed before, however rather in the context of peripheral vascular beds and during inflammation and indeed not for T cells. Tricellular junctions at the BBB are molecularly distinct and T-cell migration across tricellular BBB endothelial cell junctions already significantly contributes to T-cell diapedesis across the BBB under low inflammatory conditions. Thus, we consider our obervation as very novel.

Reviewer 1 Comments for the Author:

The authors show that T-cells prefer to cross tricellular junctions and depend on the presence of certain proteins that "build" tricellular junctions. If such proteins are absent, T-cells manage to go through transcellularly. This also indicates that blocking junction route may not be an efficient target for treatment. The work is done thoroughly, and the figures are clear presented and conclusions drawn from the results are sound. However, at the end, it is not clear how they target tricellular junctions. I miss the proof that they actually effectively targeted tricellular junctions. I have some comments and potential improvements that I have listed below.
Answer: We agree with the reviewer that using the angulin-1 and claudin-5 binding proteins provides solely indirect evidence that we truly target the tricellular or bicellular junctions, as the bining proteins may in fact engage angulin-1 and claudin-5 outside of the bona fide junctions. Performing binding studies with fluorescently tagged angubindin-1 and claudin-5 -modulator on pMBMEC monolayers suggested that both proteins induce rapid internalization of the target protein as shown in the enclosed figure taken at 2 hours after incubation.

© 2021. Published by The Company of Biologists under the terms of the Creative Commons Attribution License (https://creativecommons.org/licenses/by/4.0/).

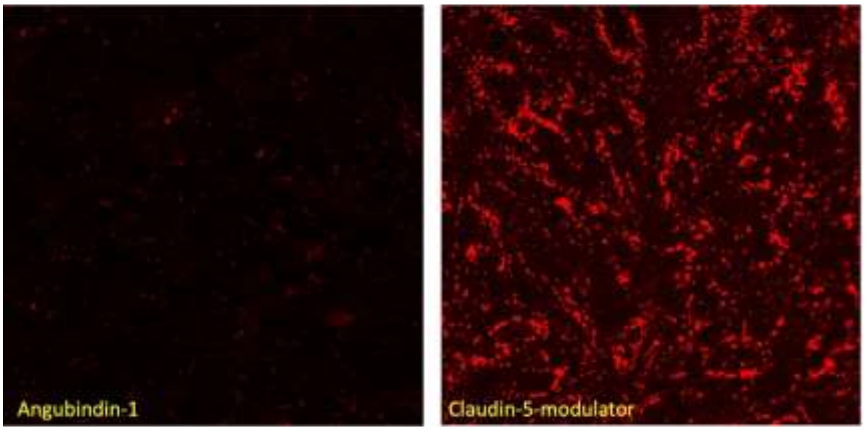

As we cannot detect subcellular localization of angulin-1 and tricellulin in pMBMEC monolayers we have significantly downtowned our manuscript in this regard. The aim of this experimental approach was solely to determine if adding proteins binding to angulin-1 or claudin-5 which have previously been shown to interfere with their appropriate localization to junctions would affect the cellular pathway of T cell diapedesis. What we do observe is that protein-targeting of both, angulin-1 and of claudin-5 does affect the cellular pathway of T-cell diapedesis across the pMBMEC monolayers. Without showing a change in the subcellular localization of angulin-1 and claudin-5 this only suggests that a lack of proper localization of these proteins to tricellular and bicellular junctions leads to the observed changes in the cellular pathway of T-cell diapedesis as observed. We have revised the manuscript accordingly.

1. In fig 1, ICAM-1 and VCAM-1 expression is shown. At higher concentrations IL1, it is clear that the expression shows a heterogenic expression pattern. The authors should at least mention this in the text.
Answer: We have edited the text accordingly and referred to previous publications that have already highlighted these characteristics of brain endothelial cells.

2. I would recommend including some arrows n figure 2, in particular when talking about certain cellular structures or organelles. Indicate the cell-cell junctions better for example.
Answer: Figure 2 has been revised also due to the request of Reviewer 2. We have introduced coloring allowing to distinguish T cells from endothelial cells and the extracellular matrix and have added arrows and asterix and have revised the figure legend accordingly.

3. I would suggest changing the abbreviation for Angulin: Ang-1, Ang-2. Ang reminds me too much to Angiopoietin….
Answer: In the revised manuscript we now refrain from using abbreviations when referring to the angulins. We have edited Figure 5 accordingly.

4. The effect on tricellular junction proteins is of interest. However, no clear functionality can be directed to it. The approach by using angubindin-1 did not really gave any satisfactory answers. Also no increase in ICAM-1, VCAM-1 etc. And yet, the authors show an increase in adhesion of T-cells (figure 6C). I am also not sure why the authors continued with this approach? What is angubindin-1 supposed to do? It did not affect permeability. Why not silencing tricellulin or overexpressing it to study its functionality better? I think that the authors should try to improve the latter part of the Ms, in particular the role of the tricellular junctions on T-cell TEM.
Answer: Knock-down or silencing approaches are unfortunately not possible in this *in vitro* BBB model, which makes use of primary mouse brain microvascular endothelial cells that are grown to confluence over 6 days and used for the respective experiments on day 7 after seeding when they have formed a tight and polarized barrier. The unique tighness of this endothelial barrier is essential to study the cellular migration pathway of T cells across the BBB under physiological flow as shown by us before (Steiner et al, JI,

© 2021. Published by The Company of Biologists under the terms of the Creative Commons Attribution License (https://creativecommons.org/licenses/by/4.0/).

2010 and Abadier et al, EJI 2015). Brain endothelial cell lines not mimicking tight barrier properties do not allow to delineate different cellular pathways of T cell diapedesis across the BBB under flow in vitro as shown by us before (Steiner et al, JI, 2010). Thus, we have to consider other approaches allowing to functionally remove junctional molecules.

Angubindin-1 is a binder of LSR/angulin-1. Angubindin-1 was previously shown to change the localization of tricellulin by removal of LSR/angulin-1 from the tricellular tight junctions (see Krug et al., J Control Release, 260, 1, 2017). We did not expect any inflammatory effects of angubindin-1 on pMBMECs thus we did also not expect upregulation of ICAM-1 or VCAM-1 on pMBMECs upon incubation with angubindin-1. Therefore, we do not have a molecular explanation for the observed increased T-cell binding to angubindin-1 pretreated pMBMECs.
We have rephrased this statement in the Results section as a suprising and unexplained finding.

The rational of incubating pMBMECs with angubindin-1 was to determine if angubindin-1 induced removal of tricellular proteins from the tricellular junctions of pMBMECs would affect the diapedesis of T cells via these tricellular tight junctions. Thus, we used angubindin-1 as a function blocking probe for the correct localization and interaction of LSR/Ang-1 in the tricellular junction. We rather observed that anbudindin-1 increased transcellular T-cell diapedesis across IL-1b^{lo} pMBMECs by reducing paracellular diapedesis but not tricellular diapedesis. This suggests that as previously shown changes in tricellular junctional architecture correlates with destabilization of tricellular and bicellular junctions (Ikenouchi et al., 2005; Krug et al., 2009) (Masuda et al., 2011) (Sohet et al., 2015).
We have improved explanations about the rational of this approach in the manuscript. We have clarified that without formal proof of a change of the subcellular distribution of junctional molecules e.g. tricellulin or angulin-1 we cannot make the statement that altered junctional architecture will affect the change in the cellular pathway of T cell diapedesis. Based on previous reports of the effects of angubindin-1 and the claudin-5 binding proteins and our previous obesrvations we think we can however at least speculate that protein-based targeting of these junctional molecules may affect the correct junctional architecture of pMBMEC tricellular and/or bicellular junctions which will lead to a change in the cellular pathway of T cell diapedesis across pMBMEC monolayers.

We have made an additional attempt to detect localization of tricellulin and angulin-1 in pMBMEC monolayers by rat-anti mouse monoclonal antibodies provided by Prof. Mikio Furuse (NIPS, Okazaki, Japan; Iwamoto, et al. 2014. *Cell structure and function*. 39:1-8). Using these antibodies we could detect localization of tricellulin and angulin-1 at the BBB in tricellular junctions in frozen brain tissue sections but we failed to obtain a specific immunostaining in pMBMEC monolayers. As we do detect specific expression of these molecules at the protein level using Western Blot we can only conclude that the available reagents cannot bind to their epitope on angulin-1 and tricellulin in pMBMEC monolayers. Thus, we agree with the Reviewer that formal proof of the correct localizatio of these proteins in pMBMEC monolayers is missing.

In order to provide additional evidence for a role of BBB tricellular junctions in T-cell diapedesis we have now performed an entirely novel set of experiments making use of our novel nanomembrane divided two-chamber ¤SiM microfluidic devices, where we cultured pMBMECs on these highly permeable nanomembranes allowing to explore the effect of abluminal (i.e. CNS derived) chemokines in a bottom chamber. These data show that chemokines may direct T cell diapedesis across pMBMECs to tricellular junctions and thus further underscored an important role of BBB tricellular junctions in T-cell diapedesis across the BBB. These novel data have been included as Figure 8 in the revised mansucript. The accompanying explanations have been integrated in Material&Methods, Results and the Discussion.

5. And in the last paragraph of the result section they state that: “To address if modulation of tricellular junctions would affect diapedesis….” But did the authors actually modulate tricellular junctions, other than IL1treatment??

© 2021. Published by The Company of Biologists under the terms of the Creative Commons Attribution License (https://creativecommons.org/licenses/by/4.0/).

Answer: As outlined in our answer to question 4 we have used angubindin-1 as a binder of LSR/Ang-1. It was previously shown that angubinin-1 inhibits correct localization of LSR/Ang-1 and tricellulin to tricellular junctions. We agree however with the Reviewer that we have no formal evidence for an angubindin-1 induced change in the junctional architecture of the tricellular junctions of the pMBMEC monolayers. This is due to the fact that although antibodies recognizing tricellulin and angulin-1 stain tricellular junctions of epithelial cells (Supplementary Figure 2) we have failed to show staining for these proteins in monolayers of pBMBMECs irrespective of the staining method used while at the same time specific detection of these junctional proteins in pMBMECs by Western Blot was readily possible (Fig. 5). As outlined above also using the monoclonal antibodies from Prof. Mikio Furuse binding to angluin-1 and tricellulin we could not detect these proteins in the pMBMEC monolayers while staining in tricellular junctions of brain endothelial cells in frozen brain sections showed specific detection as published by the Furuse laboratory (Iwamoto, et al. 2014. *Cell structure and function*. 39:1- 8). As explained above we have thus downtoned our conclusions.

6. The last statement: "…targeting tricellular junctional components in brain endothelium favors transcellular T-cell diapedesis… ", I am not convinced that they have actively targeted tricellular junctions. For this, I would suggest at least a knock down.
Answer: The Reviewer has raised an important point and we have rephrased this statement according also to our explanations given for questions 4 and 5. We have downtoned our argument such that we describe that angubindin-1 was used as a binder of LSR/angulin-1. We refrain from using the statement that we directly target tricellular junctions, but we think it is still valid to refer to angulin-1 a a "tricellular junctional component". In fact, angubindin-1 was previously shown to change the localization of tricellulin by removal of LSR/angulin-1 from the tricellular tight junctions (see Krug et al., J Control Release, 260, 1, 2017). We have no formal proof that this also occurs in our pMBMEC monolayers. However, we do see that angubindin-1 affects the cellular pathway of T-cell diapedesis across IL-1b^{lo} pMBMECs which suggests that angugindin-1 induced changes in the molecular architecture of pMBMEC junctions prohibiting paracellular T-cell diapedesis across pMBMEC monolayers under flow.

7. Minor:
• typo in abstract: junctional
• half way page 5: word missing: stimulated ECs
Answer: The mistakes have certainly been corrected.

Reviewer 2 Advance Summary and Potential Significance to Field:
Dias et al study diapedesis of T cells across cerebral endothelial monolayers under flow, a model which has consistently and in various labs delivered important insights into the molecular details of transendothelial leukocyte migration in general and key mechanistic differences between different molecular beds.
Here the authors have studied diapedesis primarily by time lapse microscopy and crucially, by painstaking, reconstructive serial block face scanning electron microscopy. They found that diapedesis location occurred paracellularly, both at bicellular and tricellular endothelial contact sites, as well as transcellularly. Additional data point to the importance of tricellular junction composition in determining the diapedesis route. Overall, it is at this point that this study falls short and does not deliver unequivocal data to support potentially important conclusions drawn. If the authors can fix that, this study will make an important contribution to how the BBB regulates immune cell infiltration, in particular how it is dependent on the presence of specialised tricellular junction proteins of the MARVEL family.
Comment: We thank the Reviewer for this overall positive statement. The criticism is very well taken and we have tried to address the concerns of the Reviewer as outlined below.

Reviewer 2 Comments for the Author:
Major comments:
The model of IL-1b(low) and (hi) is not new and is mostly well characterised. However, in the abstract and the discussion, it is claimed that results from the IL-1b(low) cells are

© 2021. Published by The Company of Biologists under the terms of the Creative Commons Attribution License (https://creativecommons.org/licenses/by/4.0/).

representative of cerebral immune cell under conditions of immune surveillance. What is the basis for likening this in vitro cell system to cerebral immune surveillance (for which the role of the microvasculature, to the best of my knowledge, is still not unequivocally demonstrated)?

Answer: Comparative anlaysis of IL-1b^{lo} and IL-1b^{hi} pMBMECs has indeed been described by us before (Abadier, et al., Eur J Immunol. 2015 Apr;45(4):1043-58). Here we have used these different inflammatory conditions for the first time for an ultrastructural analysis of T-cell diapedesis across pMBMECs as *in vitro* model of the BBB under physiological flow. Data from us and others provide accumulating evidence that under low to no infllammatory conditions T- cell migration across the BBB occurs rather at the endothelial cell junctions. (Abadier, et al., Eur J Immunol. 2015 Apr;45(4):1043-58; Lutz et al, Cell Rep. 2017 Nov 21;21(8):2104-2117). The Reviewer is absolutely right that our data only show conditions of low and no inflammation. This is due to the fact that in the absence of any inflammatory stimulus the numbers of T cells interacting with the pMBMECs would be too low to perform a SBF-SEM study. We have thus corrected the entire text such that when referring to our data we solely refer to low and high inflammatory conditions and then only speculate in the discussion that intact molecular architecture of the paracellular and tricellular junctions is important for guiding T cells trough these junctional pathways.

Figure 1A: This figure is claimed to show unaltered TJ distribution. However, in IL1b treated cells TJ continuity appears impaired. In addition, judging by the nuclear stain, some of the IL1b-stimulated monolayers appear to consist of piled up endothelial cells.
Answer: The critique of the Reviewer is very well taken. pMBMECs grow as primpary cells within one week to a confluent monolayer and the endothelial cells are strictly contact inhibited. The “piled up” nuclei this Reviewer has criticized are due to pericytes that are a variable and minor contamination in these cultures and were visible in the figure. We appreciate that this may cause confusion. Based on the critique of this Reviewer we have therefore performed additional immunostainings on non-stimulated, IL-1b^{lo} and IL-1b^{hi} pMBMECs. Blinded experimenters have analysed the images and we came to the conclusion namely that there is no obvious difference in junctional continuity between NS, IL-1b^{lo} and IL-1b^{hi} pMBMECs with respect to claudin-5, ZO- 1 and VE-cadherin. Occluding staining could be distinguished between NS and IL-1b^{lo} and IL- 1b^{hi} pMBMECs where some dotty occludin staining is visible outside of the junctional localization in IL-1b but not in NS pMBMEC monolayers. There is in addition a trend towards increased F-actin stress fiber formation in IL1b-stimulated pMBMEC monolayers. In the revised Figure 1A we have now included higher maginifcation images for occludin allowing for a better judgement of its junctional and non-junctional localization and have included an additonal immunostaining for F-actin in VE-cadherin-GFP^{+} pMBMECs visualizing potential stress-fiber formation and an adherens junctional component.

Robustness of permeability measurements:
The authors have shown in a previous publication (Abadier et al) that barrier properties are altered in response to IL1b pre-treatment. In the present dataset, there is a trend, which is not borne out statistically. I propose that the authors alter their method to reduce the assay error or increase the number of independent repeats to unequivocally establish if these trends are real. While this is not so much a problem in figure 1, it is much more so for statements on barrier integrity following intervention with angubindin or the claudin modulators (Figure 6).
Answer: The *in vitro* permeability of the pMBMEC monolayers was assessed by measuring the clearance of two fluorescent tracers which is THE state-of-the-art methodology and superior to single end-point diffusion studies. We have now elaborated on this methodology in Material and Methods and provided all the original references allowing direct access to the calculation of the permeability coefficient for the specific tracers based on this clearance principle. We hope this clarifies the usefulness and vailidity of this method. We also specifically used small molecular tracers to determine subtle differences in permeability of pMBME monolayers.

Our present data exactly reproduce our previoius findings already reported in *Abadier et*

© 2021. Published by The Company of Biologists under the terms of the Creative Commons Attribution License (https://creativecommons.org/licenses/by/4.0/).

al, EJI, 2015. This previous study found a significant decrease of the TEER in IL-1^{hi} versus TNF-¤- stimulated pMBMECs as measured by impedance spectroscopy. At the same time there was a trend towards increased permeability to small molecular tracers across IL-1^{hi} versus TNF-¤- stimulated pMBMECs, but this difference was also in the previous study not quite significant.

Our present study confirms over a total of 4 assays with triplicates for each value and for 2 different tracers that also when comparing IL-1^{lo} and IL-1b^{hi} pMBMECs that there is a trend for increased permeability of these tracers across IL-1b^{hi} versus IL-1b^{lo}pMBMECs. These data therefore are exactly in line with our previous observations.

Including additional TEER measurements in the revised manuscript would have been optimal. Unfortunatley we had to replace our CellZScope and due to the Covid-19 pandemic this has been delayed.

We have thus downtoned our statements on barrier properties of IL1b^{hi} versus IL1^{lo} pMBMECs throughout the manuscript.

In the discussion the authors state that they "found that decreased expression of tricellulin and LSR/angulin in IL-1bhi) stimulated pMBMECs was increased permeability to small molecules". Where is statistically robust data illustrating this shown?
Answer: As outlined above we have downtoned our wording with respect to an increase of permeability of IL1b^{hi} versus IL1b-1^{lo} pMBMEC monolayers as we only see a trend that is not quite statistical significant difference of IL1^{hi} versus IL-1^{lo} pMBMECs in our assays.

Permeability (flux) measurements were carried out using cells cultured in transwells in the absence of flow. Whilst these are conditions considerably different to those used for the transmigration assays, I do not consider this a problem per se. However, in the discussion the authors claim that the reason for not reproducing published effects of CLDN-5 modulation (Neuhaus et al) is due to their system not allowing access to the basal side of the ECs, which clearly cannot be true.
Answer: Our permeability assays show that pMBMECs establish a very tight cellular barrier to small molecular tracers. As the pMBMECs establish this tight and polarized barrier as a primary cell culture without the need of co-cultures with pericytes or astrocytes we can avoid using sophisticated microfluidics devices with two chambers to assess T-cell interaction with the BBB under physiological flow by in vitro live cell imaging. In our standard microfluidic device there is no lower chamber. Thus, in contrast to the previously published studies we added the claudin- targeting proteins only to the luminal side and not in addition to the abluminal side. The previous studies have shown claudin-5 modulation in other in vitro BBB models to be different when adding the claudin-5 targeting proteins from the abluminal and luminal side. We thus consider it acceptable to speculate in the discussion that our assay system might not have shown an effect on permeability due to only the luminal incubation with claudin-5 targeting proteins. The Reviewer is however right, numerous other reasons could be considered. We have now also mentioned that of course expression levels of claudin-5, junctional assembly and architecture in the pMBMECs could be different compared to the previous in vitro BBB models used.

EM and time lapse imagery:
Figure 2: The authors have made a great effort to gather EM data on transmigrating T cells. However, more illustrating details should be shown so that the amount of ultrastructural detail can be fully appreciated. For instance, more arrowing of key features and colour shading of the two cell types is often used for similar datasets.
Answer: We have color shaded the figures as requested and have added additional arrows to better highligh the subcellular structures referred to.

Additionally, the authors use these images to derive quantitative data on paracellular and transcellular transmigration events. None of the images shown in this figure allow such a categorisation and images of representative examples must be shown.
Answer: As corretly pointed out by the Reviewer it is rather the “transversal” sections

© 2021. Published by The Company of Biologists under the terms of the Creative Commons Attribution License (https://creativecommons.org/licenses/by/4.0/).

shown in Figure 3A not the “coronal” sections shown in Figure 2 that were used to quantify the cellular pathway of T-cell diapedesis at the ultrastructural level. We hope that additional coloring of the figures and adaption of the Figure legends have clarified the open questions in this regard.

Figure 3. Panel A: Again, additional colour shading of the T and EC cell would be of great help to fully appreciate the images.
Answer: Figure 3 A has been color-coded as requested. These 3 examples do show transcellular, paracellular and tricellular diapedesis. At his point we have to thank the Reviewer for requesting to color these figures, which led to the thorough re-analysis of the respective pictures and the replacement of the example for transcellular diapedesis to show a much clearer example for this diapedesis pathway.

Additionally, what is the proof that the T cell purported to represent a paracellular transmigration event is not ultimately diapedesing through the nearby junction?
Answer: We are not entirely sure if we understand the question of the Reviewer. We have defined diapedesis of a T cell when the T-cell nucleus was inserted though the endothelial monolayer as previously described by Barzilai et al., 2017 and outlined in the manuscipt. The examples shown in Figures 2 and 3A do show how we identified the cellular pathway of T-cell diapedesis. In these transversal sections one can always see the T-cell nucleus surrounded by T-cell cytoplasm inserted through the endothelial monolayer. We hope the coloring of the examples highlighting the T cell nucleus in a different color as the T cell cytoplasm have made this more clear.

Given that these EM pictures only show isolated time points, it is necessary that the authors demonstrate that the cells shown fulfil the criteria of "at least part of their nucleus inserted through the pMBMEC monolayer".
Answer: As outlined above this is exactly what is shown in Figures 2D and 3A – examples for T cells having inserted their nucleus across the endothelial monolayer at the respective locations. We hope the coloring of the examples highlighting the T cell nucleus in a different color as the T cell cytoplasm have made this more clear.

Panel B: The remainder of the manuscript relies on diapedesis differences across IL-1b(low) and (high) cells. Some kind of statistical analysis must be undertaken to demonstrate that this does not represent a random segregation of observations.
Answer: In order to show that T cell diapedsis across tricellular or bicellular junctions or across the endothelial body is not random we have established a base-line mode corresponding to a situation where the T cells would ramdomly choose a site for diapedesis. In this model the probability of T-cell diapedesis via a certain site of the pMBMEC monolayer would be directly proportional to their occurrence in the monolayer. We have used a boundary segmentation approach allowing to segment cell boundaries, detect cellular junctions and assign a radius to these jucntions including the tricellular junctions. Using this approach even if assigning an unrealistic radius of 6 □m to cellular junctions, T-cell diapedesis across tricellular junctions would only reach a fraction of 25%. We have included description of this approach in the results and have added the visualization of this segmentation approach as Supplementary Figure 2.

We have additionaly included pstatistical analysis of the *in vitro* live cell imaging studies shown in Figures 4, 5 and 6 with the control datasets investigated in the absence of added proteins.
These data have now been included as Figure 8A. Analysis these several 100 diapedesis events on $IL\text{-}1b^{hi}$ or $IL\text{-}1b^{lo}$ pMBMECs underscore that T-cell diapedesis via tricellular junctions is not a random segregation event and the significant increase in transcellular T-cell diapedesis across $IL1^{bhi}$ stimulated pMBMECS, when compared to the $IL1b^{lo}$ pMBMECs.

Figure 4: There are several problems with the imaging data shown here. What is the line separating different parts of exposure in the VEcad images? Without outlining the position and circumference of the T cells in the VEcad images, it is not possible to fully determine where exactly diapedesis occurs.

© 2021. Published by The Company of Biologists under the terms of the Creative Commons Attribution License (https://creativecommons.org/licenses/by/4.0/).

Answer: To image sufficient T-cell diapedesis events over the entire pMBMEC monolayer and thus to reduce the number of mice to be sacrificed for pMBMEC isolations we decided to image the entire field of view with high resolution. To achieve this goal we subdivided our region of interest into multiple smaller images, referred to as tiles. These tiles are imaged individually and then combined via stitching to a larger overwiew as shown in the example below. Thus, the lines visible in Figure 4B and C are due to the fact that the examples highlighted here takes place close to a border where the individual images were stiched together. We have elaborated on this method in Materlial and Methods and added an explanation in the Figure legend and hope this clarifies the issue.

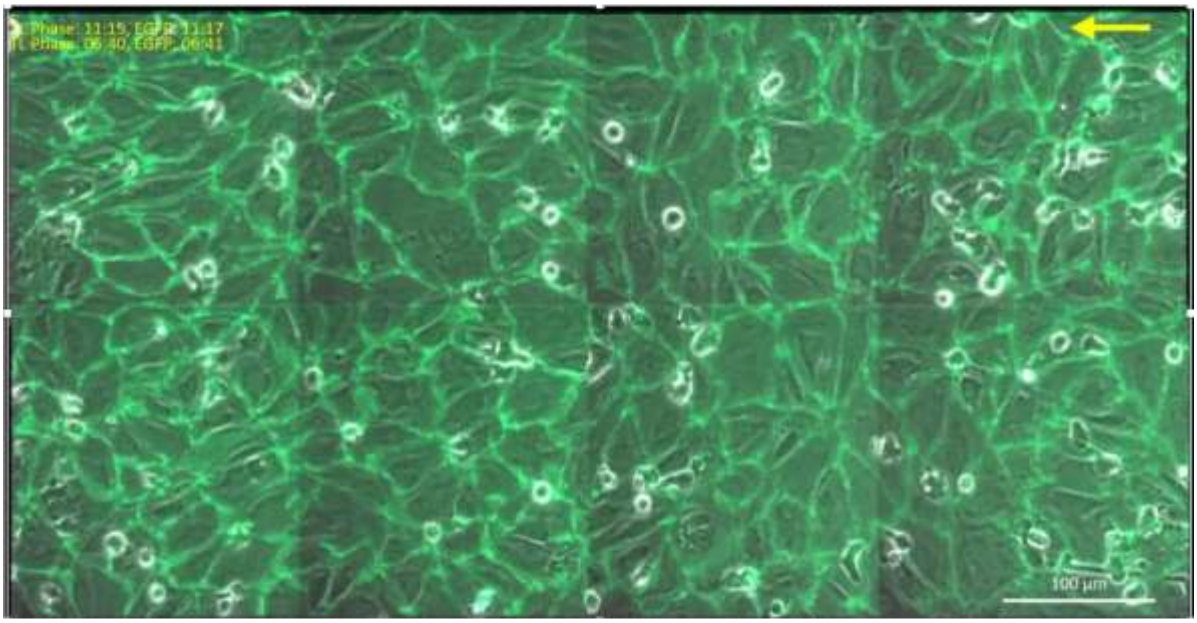

We have previously used the VE-cadherin-GFP pMBMECs to analyze the cellular pathway of T cell diapedesis across pMBMEC monolayers (Abadier et a., EJI 2015, Wimmer et al, Front Immunol., 2019). Separation of the junctional green fluorescence allows to unequivocally identify the cellular pathway of T-cell diapedesis. The circumference of the cell does not allow to understand where the T cell crosses the pMBMEC monolayer. This is why we have specifically added the yellow arrow pointing to the exact same spot of T-cell diapedesis in the same field of view in the fluorescence and phase contrast images. The phase contrast pictures of the identical frame below the fluorescent picture do show the outline of the T cell on top of the pMBMEC monolayer exactly as requested by the Reviewer. This highlights the T cell in the process of diapedesis but it is the yellow arrow that shows the correct site of diapedesis below the T cell body. We have rephrased the figure legend to clarify these issues.

There are many instances where the authors fail to reproduce important prior observations in their experimental cells system and this impacts the overall value of the study (see also above comment on barrier measurement):
Answer: We do not quite understand the basis of this critique as our current study reproduces in full all our previous and published observations on the effect of high and low concentrations of IL-1b on pMBMECs with respect to regulation of adhesion molecules, changes in permeability as well as in the ratios of junctional versus transcellular T-cell diapedesis.

Other studies have neatly shown the localisation of MARVEL proteins to tricellular junctions in cultured endothelial cells or intact blood vessels. Thus it is insufficient for the authors to simply state that they have not been able to achieve staining for tricellulin or angulins, in particular since the reduced level of expression of these proteins as measured by WB is mild and may well be due to their complete

© 2021. Published by The Company of Biologists under the terms of the Creative Commons Attribution License (https://creativecommons.org/licenses/by/4.0/).

disappearance at a small subset of tricellular contact points (which ultimately no longer serve as diapedesis sites). Given that the quantitative differences the authors base their conclusions on, are subtle, a more in-depth analysis of MARVEL protein localisation with respect to diapedisis routes is required. An even more damning interpretation of the data shown could be that, whilst these MARVEL proteins are expressed, they fail to localise appropriately in these mouse endothelial cell cultures, which would invalidate most of the conclusions drawn.

Answer: We are not aware of any study that has successfully shown stainig for angulins and/or tricellulin in tricellular junctions of a mouse *in vitro* BBB model. We have in fact obtained the monoclonal pan-anti mouse angulin-1 and tricellulin antibodies from Prof. Mikio Furuse and have repeated stainings on pMBMEC monolayers using differen fixation protocols as well as on frozen brain tissue sections. We can nicely reproduce the stainings showing localization of tricellulin and angulin-1 in tricellular junctions of endothelial tricellular junctions in mouse brain tissue sections but we do not see specific stainings in our pMBMEC monolayers. Please note we do see specific staining for these tricellular proteins in mouse epithelial cell cultures as shown in Supplementary Figure 2. Thus, we do not "simply state" that we cannot show subcellular localization of tricellulin and angulin-1 in our pMBMEC monolayers but rather do see this as a limitation of the present study, as correctly pointed out by this Reviewer.

Neverthesless the additional experiments and analyses performed strongly support the role of tricellular junctions in mediating T-cell diapedesis across the BBB. We have thus revised the entire manuscript according to these additional insights and better highlight that we have no evidence for a role for angulin-1 and tricellulin in this process but that the molecular underpinnings of T cell diapedesis via tricellular junctions at the BBB remain to be explored.

The authors claim that expression levels of MARVEL proteins are important for the location of diapedesis. The argument for using proteins/peptides interfering with their function is justified by the lethality of ko mice. Why have MARVEL protein expression not been modulated using siRNA (which deliver a measurable biochemical endpoint demonstrating successful intervention)?

Answer: As outlined above based on our novel data and analyses and the limitations of our study we have revised the entire manuscript such that we do not imply a direct role for appropriate localization of angulin-1 and tricellulin at the tricellular junctions for T-cell diapedesis across these tricellular junctions.

The *in vitro* model of the BBB used in this study is established from primary mouse brain microvascular endothelial cells that are grown to confluence over one week prior to be used in the respective assays. This in vitro BBB model does not allow for efficient transduction and thus overexpression or knock-down approaches. At the same time the superior barrier characteristics of pMBMECs are necessary to model the cellular pathway of T-cell diapedesis across the BBB under physiological flow as shown by us before (Steiner et al., JCBFM, 2009, Steiner et al, JI 2010). Thus knock-down or overexpression approaches are not possible in this experimental setup.

Overall the protein/peptide tools used to modulate MARVEL protein functions have been well characterised in other, published datasets but not here. The authors themselves concede that e.g. CLDN interference may depend on access to the basal side of the endothelium. Proof of successful intervention must be provided.

© 2021. Published by The Company of Biologists under the terms of the Creative Commons Attribution License (https://creativecommons.org/licenses/by/4.0/).

Answer: We have incubated pMBMEC monolayers with fluorescently labeled angubindin-1 and claudin-5 binding protein, which has allowed us to determine the rapid uptake of these proteins by the pMBMECs within 2 hours by confocal microscopy. As shown here in the Figure provided for the Reviewer this suggests that angulin-1 and claudin-5 are prohibited from properly integrating into the tricellular and bicellular junctions as described before. We have included mention of this observation as data not shown in the revised manuscipt.

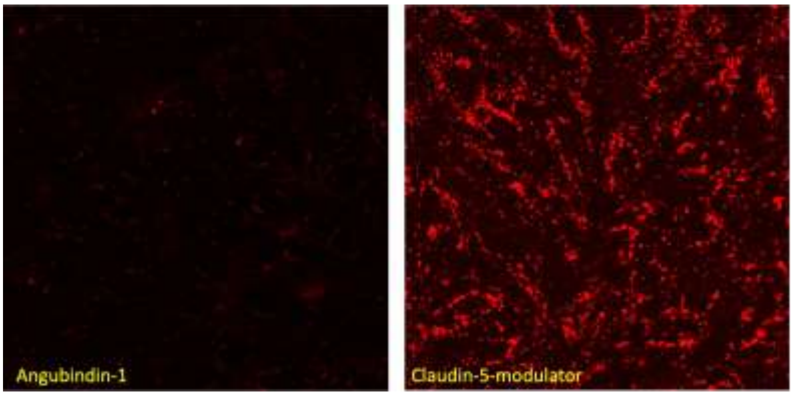

Overall, the manuscript is long-winded with too many sections inserted as important arguments in the absence of clear data. The authors should stick to the main message and produce a clear, linear narrative to convey the message.
Answer: The manuscript has been extensively revised, shortened and focused.

The authors use many very definitive adjectives to describe their findings and emphasise their impact. However, these should be adapted to reflect findings more realistically. 'Novel' is used for the observation of migration at tricellular junctions. However, as covered in detail in the discussion, this phenomenon has been described before. I understand that the authors believe that their data show clear differences between diapedesis at the BBB and the periphery, however, to reach this conclusion more molecular detail must be shown (as discussed above).
Answer: We have added a novel set of experiments that further underscore the role of tricellular junctions in pMBMEC monolayers in T-cell diapedesis (Fig. 8, Supplementary Figure 2). We have downtowned our manuscript as requested. Given the fact that tricellular junctions of the BBB are molecularly distinct from those of peripheral vascular beds and that we observe T-cell diapedesis across tricellular junctions of the BBB under low inflammatory conditions we still think this is an entirely novel observation. We have clarified that the molecular mechanisms involved in this process remain to be investigated.

Diapedesis at tricellular junctions is also noted as the 'preferred route', however it makes up for less than 50% of all migration events, and in the absence of any meaningful statistical evaluation may be similar to migration at bicellular junctions.
Answer: As outlined above we have used a boundary segmentation approach on the pMBMEC monolayer to show that T-cell diapedsis across tricellular junctions is not random. We have established a base-line mode corresponding to a situation where the T cells would ramdomly choose a site for diapedesis. In this model the probability of T-cell diapedesis via a certain site of the pMBMEC monolayer would be directly proportional to their occurrence in the monolayer. We have used the boundary segmentation approach allowing to segment cell boundaries, detect cellular junctions and assign a radius to these junctions including the tricellular junctions. Using this approach even if assigning an unrealistic radius of 6 □m to cellular junctions, T-cell diapedesis across tricellular junctions would never by beyond 25%. We have included description of this approach in the results and have added the visualization of this segmentation approach as Supplementary Figure 2.

Minor:
In the discussion the authors describe tricellulin as a MARVEL family protein, however

© 2021. Published by The Company of Biologists under the terms of the Creative Commons Attribution License (https://creativecommons.org/licenses/by/4.0/).

angulins are also part of this family.
Answer: MARVEL is a domain with a four transmembrane-helix architecture that has been identified in proteins of the myelin and lymphocyte (MAL), physins, gyrins and occludin families. The tight junction associated MARVEL protein (TAMP) family comprises tricellulin, occludin and MARVEL domain-containing 3 (marveld3). In contrast, the angulins are type I transmembrane protains with an exctracellular Ig like domain. Thus, angulins do not belong to the MARVEL family.

Whilst very interesting, the reconstruction work illustrating nuclear deformation in T cells during diapedesis does not serve any purpose with regard to the message of this manuscript.
Answer: We consider this dataset as highly informative to underscore that the cellular pathway of T-cell diapedesis across the pMBMEC monolayer is not due to specific T cell nuclear shape changes and would thus like to keep this as supplementary information.

We do hope to have addressed all queries of the Reviewers in a satisfactory manner and would be very happy if our revised manuscript was accepted for publication in the *Journal of Cell Science*.

Sincerely
Dr. rer. physiol. Britta Engelhardt
Professorin für Immunbiologie

Second decision letter

MS ID#: JOCES/2020/253880

MS TITLE: Brain endothelial tricellular junctions as novel sites for T-cell diapedesis across the blood-brain barrier

AUTHORS: Mariana Castro Dias, Adolfo Odriozola Quesada, Sasha Soldati, Fabio Boesch, Isabelle Gruber, Tobias Hildbrand, Derya Soenmez, Tejas Khire, Guillaume Witz, James L McGrath, Joerg Piontek, Masuo Kondoh, Urban Deutsch, Benoit Zuber, and Britta Engelhardt
ARTICLE TYPE: Research Article

We have now reached a decision on the above manuscript.

To see the reviewers' reports and a copy of this decision letter, please go to: https://submit-jcs.biologists.org and click on the 'Manuscripts with Decisions' queue in the Author Area. (Corresponding author only has access to reviews.)

As you will see, both reviewers are satisfied with the response to their concerns, but reviewer 2 has a couple of minor changes that should be addressed. These do not involve new experimentation, and as such, should be easily achieved. I look forward to to seeing the revised version.

We are aware that you may be experiencing disruption to the normal running of your lab that makes experimental revisions challenging. If it would be helpful, we encourage you to contact us to discuss your revision in greater detail. Please send us a point-by-point response indicating where you are able to address concerns raised (either experimentally or by changes to the text) and where you will not be able to do so within the normal timeframe of a revision. We will then provide further guidance. Please also note that we are happy to extend revision timeframes as necessary.

Please ensure that you clearly highlight all changes made in the revised manuscript. Please avoid using 'Tracked changes' in Word files as these are lost in PDF conversion.

© 2021. Published by The Company of Biologists under the terms of the Creative Commons Attribution License (https://creativecommons.org/licenses/by/4.0/).

I should be grateful if you would also provide a point-by-point response detailing how you have dealt with the points raised by the reviewers in the 'Response to Reviewers' box. Please attend to all of the reviewers' comments. If you do not agree with any of their criticisms or suggestions please explain clearly why this is so.

Reviewer 1

Advance summary and potential significance to field

As stated before, this study is a great addition to the current field of T-cell transmigration across BBB.

Comments for the author

The authors have addressed all my comments. I in particular appreciate the additional two-chamber SIM microfluidic experiments. Well done.

Reviewer 2

Advance summary and potential significance to field

There is still much we do not know about the molecular mechanisms of leukocyte transmigration across blood neural barriers, including potential differences to transmigration events in the periphery. It has long been hypothesised that differences in junctional complement and organisation account for differences in how leukocytes diapedese across different vascular beds. By exploiting differences in junctional functions and by meticulously imaging diapedesis events by EM the authors have been able to deliver very good evidence in support of this hypothesis, namely that tricellular junction function is instrumental for paracellular diapedesis across the BBB in vitro. I believe that this constitutes a signficant advance in the field.

Comments for the author

Overall, the authors have addressed my concerns adequately and I find this manuscript much improved. I commend the authors for their meticulous work, in particular, the beautiful job they have done with the EM imagery.
I am now also entirely satisfied that the conclusions match the data.

There are some remaining minor points, which the authors may want to address:
1. Overall the text is still very ponderous at places and as such distracts from the elegance of many of the data. As an example, I do not think it is necessary to keep on repeating the conditions used for diapedesis.
This statement and the reference (Barzilai et al 2017) appear three times on page 5 alone.
2. Whilst the pseudo-colouring of the EM imagery in Figure delivers much more insight, even to the technically less well versed, I am not sure everybody would be able to follow the observations and arguments made regarding organelle/uropod positioning. I think in this regard this figure could do with further clarifying annotations.
3. I believe there must be a mistake in the labelling (both in the text and the legends) regarding Figure 8A and B. It should be clearly pointed out how this data set is (technically) different from any of the previous.
4. It is not of great importance, but given their response the authors must have misunderstood my question regarding the determination of the diapedesis pathway: I previously questioned how the methodology used can distinguish between a T cell migrating exclusively through a junction or alternatively a T cell that has undergone transcellular diapedesis immediately adjacent to a junction. I understand that the analysis of many transversal sections will allow this distinction and was wondering if every definitive assigned diapedesis event was analysed in such a detailed way.
5. In their rebuttal, the authors make the very important point that knockdowns/transfections are not feasible in the culture system they used. Since this is a limitation that restricts many BBB model analyses, I would appreciate if the authors made that very point also in the manuscript.

© 2021. Published by The Company of Biologists under the terms of the Creative Commons Attribution License (https://creativecommons.org/licenses/by/4.0/).

6. The manuscript still contains many typos, which I am sure will be amended in proof.

Second revision

Author response to reviewers' comments

Submission of our revised manuscript JOCES/2020/253880 entitled “Brain endothelial tricellular junctions as novel sites for T-cell diapedesis across the blood-brain barrier» for publication in the Journal of Cell Science

Dear Dr. Billadeau

Please find enclosed our revised manuscript entitled “Brain endothelial tricellular junctions as novel sites for T-cell diapedesis across the blood-brain barrier” for publication in the Journal of Cell Science.

We thank both Reviewers for their positive feed-back to our revised manuscript. We have addressed the minor issues requested by Reviewer 2 as outlined in our point-by-point reply below. Changes in the revised manuscript are highlighted in blue.

1. Overall the text is still very ponderous at places and as such distracts from the elegance of many of the data. As an example, I do not think it is necessary to keep on repeating the conditions used for diapedesis. This statement and the reference (Barzilai et al 2017) appear three times on page 5 alone.
Answer: The manuscript was edited one more time to avoid remaining redundancies and shorten where possible. We agree that some paragraphs need concentrated reading due to the complicated experimental setup or the precision needed to explain the precise experimental workflow. We hope that the revised manuscript now has a better flow.

2. Whilst the pseudo-colouring of the EM imagery in Figure delivers much more insight, even to the technically less well versed, I am not sure everybody would be able to follow the observations and arguments made regarding organelle/uropod positioning. I think in this regard this figure could do with further clarifying annotations.
Answer: We have added additional annotations and further detailed the Figure legend to further improve clarity of this figure.

3. I believe there must be a mistake in the labelling (both in the text and the legends) regarding Figure 8A and B. It should be clearly pointed out how this data set is (technically) different from any of the previous.
Answer: We thank the Reviewer for pointing out this oversight. The Figure legend of Figure 8 and the text of the Results has been corrected accordingly.

4. It is not of great importance, but given their response the authors must have misunderstood my question regarding the determination of the diapedesis pathway: I previously questioned how the methodology used can distinguish between a T cell migrating exclusively through a junction or alternatively a T cell that has undergone transcellular diapedesis immediately adjacent to a junction. I understand that the analysis of many transversal sections will allow this distinction and was wondering if every definitive assigned diapedesis event was analysed in such a detailed way.
Answer: The cellular pathway of T cell diapedesis across the pMBMEC monolayers in the SBF-SEM dataset was indeed analysed by visual inspection of each T cell found at the frontal and transveral plane. We have added additional explanations to the Results and Methods.

5. In their rebuttal, the authors make the very important point that knockdowns/transfections are not feasible in the culture system they used. Since this is a limitation that restricts many BBB model analyses, I would appreciate if the authors made that very point also in the manuscript.

© 2021. Published by The Company of Biologists under the terms of the Creative Commons Attribution License (https://creativecommons.org/licenses/by/4.0/).

Answer: We have added an explanation of the limitations of this in vitro BBB model in Material and Methods.

6. The manuscript still contains many typos, which I am sure will be amended in proof.
Answer: The manuscript has been edited once more to remove any typing errors.

We do hope to have addressed all queries of the Reviewer in a satisfactory manner and would be very happy if our revised manuscript was now accepted for publication in the Journal of Cell Science.

Sincerely
Dr. rer. physiol. Britta Engelhardt
Professorin für Immunbiologie

Third decision letter

MS ID#: JOCES/2020/253880

MS TITLE: Brain endothelial tricellular junctions as novel sites for T-cell diapedesis across the blood-brain barrier

AUTHORS: Mariana Castro Dias, Adolfo Odriozola Quesada, Sasha Soldati, Fabio Boesch, Isabelle Gruber, Tobias Hildbrand, Derya Soenmez, Tejas Khire, Guillaume Witz, James L McGrath, Joerg Piontek, Masuo Kondoh, Urban Deutsch, Benoit Zuber, and Britta Engelhardt
ARTICLE TYPE: Research Article

I am happy to tell you that your manuscript has been accepted for publication in Journal of Cell Science, pending standard ethics checks.

© 2021. Published by The Company of Biologists under the terms of the Creative Commons Attribution License (https://creativecommons.org/licenses/by/4.0/).
